# Decontamination of water co-polluted by copper, toluene and tetrahydrofuran using lauric acid

**DOI:** 10.1038/s41598-022-20241-4

**Published:** 2022-09-22

**Authors:** Laura Earnden, Alejandro G. Marangoni, Thamara Laredo, Jarvis Stobbs, Tatianna Marshall, Erica Pensini

**Affiliations:** 1 School of Engineering, University of Guelph, Room 2525 Richards Bld., 50 Stone Road East, Guelph, ON N1G 2W1 Canada; 2 Food Science Department, University of Guelph, 50 Stone Road East, Guelph, ON N1G 2W1 Canada; 3 Chemistry Department, Lakehead University, 500 University Ave, Orillia, ON L3V 0B9 Canada; 4 Canadian Light Source Synchrotron, 44 Innovation Boulevard, Saskatoon, SK S7N 2V3 Canada

**Keywords:** Chemical engineering, Pollution remediation, Chemistry, Engineering

## Abstract

Co-contamination by organic solvents (e.g., toluene and tetrahydrofuran) and metal ions (e.g., Cu^2+^) is common in industrial wastewater and in industrial sites. This manuscript describes the separation of THF from water in the absence of copper ions, as well as the treatment of water co-polluted with either THF and copper, or toluene and copper. Tetrahydrofuran (THF) and water are freely miscible in the absence of lauric acid. Lauric acid separates the two solvents, as demonstrated by proton nuclear magnetic resonance (^1^H NMR) and Attenuated Total Reflection-Fourier Transform Infrared Spectroscopy (ATR-FTIR). The purity of the water phase separated from 3:7 (v/v) THF:water mixtures using 1 M lauric acid is ≈87%v/v. Synchrotron small angle X-Ray scattering (SAXS) indicates that lauric acid forms reverse micelles in THF, which swell in the presence of water (to host water in their interior) and ultimately lead to two free phases: 1) THF-rich and 2) water-rich. Deprotonated lauric acid (laurate ions) also induces the migration of Cu^2+^ ions in either THF (following separation from water) or in toluene (immiscible in water), enabling their removal from water. Laurate ions and copper ions likely interact through physical interactions (e.g., electrostatic interactions) rather than chemical bonds, as shown by ATR-FTIR. Inductively coupled plasma—optical emission spectrometry (ICP-OES) demonstrates up to 60% removal of Cu^2+^ ions from water co-polluted by CuSO_4_ or CuCl_2_ and toluene. While lauric acid emulsifies water and toluene in the absence of copper ions, copper salts destabilize emulsions. This is beneficial, to avoid that copper ions are re-entrained in the water phase alongside with toluene, following their migration in the toluene phase. The effect of copper ions on emulsion stability is explained based on the decreased interfacial activity and compressional rigidity of interfacial films, probed using a Langmuir trough. In wastewater treatment, lauric acid (a powder) can be mixed directly in the polluted water. In the context of groundwater remediation, lauric acid can be solubilized in canola oil to enable its injection to treat aquifers co-polluted by organic solvents and Cu^2+^. In this application, injectable filters obtained by injecting cationic hydroxyethylcellulose (HEC +) would impede the flow of toluene and copper ions partitioned in it, protecting downstream receptors. Co-contaminants can be subsequently extracted upstream of the filters (using pumping wells), to enable their simultaneous removal from aquifers.

## Introduction

Industrial activities release water-soluble, toxic heavy metals in groundwater, including lead, chromium, arsenic, zinc, cadmium, mercury and copper^1,2^. Copper is used in fertilizers and pesticide sprays, building materials, and agricultural and municipal waste, causing high copper concentrations in groundwater^1^. Hydrocarbons are also widely used in industrial processes and they are amongst the most common groundwater pollutants^3^. THF is a groundwater and industrial wastewater contaminant, because it used to produce pharmaceutical and pesticide intermediates^4,5^. Heavy metals, hydrocarbons and water-miscible organic solvents (e.g., dioxane or THF) are often present as co-contaminants on industrial sites^6–11^.

Treatment of heavy metals includes electrokinetic remediation^12–16^, removal using nanoparticles^17^ and soil flushing with additives which facilitate the solubilization and extraction of heavy metals through pump and treat^1^. Pump and treat extracts pollutants using pumping wells, treats groundwater ex situ and finally reinjects it after treatment^18^. As an example, ethylenediaminetetraacetic acid (EDTA) has been used to remediate copper in conjunction with pump and treat^19^. Our previous study used sodium lauroyl lactylate (SLL) for the same purpose^20^. These approaches do not enable the simultaneous removal of heavy metals and co-contaminants such as miscible solvents, of which THF is an example.

Common remedial approaches to target hydrocarbon contamination include chemical methods (e.g., chemical degradation using Fenton’s reagents^21,22^, oxidizing emulsifiers^23^, slow release oxidizer rods^24^, oxidizing nanoparticles^25^, ferrate^26^ and ozone sparging^27^), phytoremediation for shallow contaminants^28^, microwave heating^29^, pump and treat^30^, surfactant flushing^31,32^ and surfactant enhanced oxidation^33^, bioremediation using bacteria^34,35^, smouldering^36^ and thermal treatment^37^. Electrokinetic remediation of hydrocarbons has also been proposed, for instance in conjunction with surfactants^38,39^. Hydrocarbons can migrate during their remediation, positing risks to downstream receptors^40–42^. This risk also exists for heavy metals.

Our previous research focused on immobilizing Cr(VI) during its reduction to less toxic Cr(III)^43^, and barriers can mitigate hydrocarbon migration during treatment^44^. Barriers can be non-reactive^18^ or reactive^45–50^. In our previous studies focusing on hydrocarbons we also developed semi-permeable filters that could be injected for facile installation^51^. These filters enabled water flow, while retaining hydrocarbons^51^. While useful to prevent hydrocarbon migration, these filters could not simultaneously retain heavy metals.

Organic solvents miscible in water can be removed from wastewater using biological treatment^52–54^, chemical methods (e.g., advanced oxidation processes^55^) or pervaporation^56,57^. Biological treatment requires that pollutant concentrations are below levels at which they are toxic to the bacteria. Advanced oxidation processes are useful but require potentially hazardous chemicals and pervaporation is energy intensive.

Moreover, separation between water and organic solvents can be achieved by enhancing the organic solvent hydrophobicity (i.e., strengthening the organic molecule network at the expense of water-solvent interactions). For instance, a study showed that temperature could be tuned to decrease the ionicity of the lidocaine-oleic acid phase in a mixture of water, lidocaine and oleic acid, thereby separating it into a water phase and a lidocaine-oleic acid phase^58^. It would be advantageous to achieve separation without temperature cycles. Previous studies separated water from water-miscible solvents using salts^59,60^, sugars^61,62^ and polyols^63^, and surfactants such as sodium lauroyl lactylate (SLL)^64^. These compounds hydrogen bond (H-bonds) with water, thereby hindering water-solvent interactions^64–69^. In these previous studies, copper ions were not separated from water alongside with THF.

Liquid–liquid extraction has been applied to other contaminants such as antibiotics, pesticides and hydrocarbons, to facilitate their detection through analytical methods^70–72^, but the simultaneous separation of THF and metal ions (e.g., copper) was not previously achieved in the context of water purification. Here, we use for the first time lauric acid to simultaneously separate THF from water and induce copper ion migration in the THF phase, at ambient temperature (e.g., 20 °C). The proposed approach has potential applications to treat wastewater, surface water or groundwater, for instance following its extraction from polluted aquifers through pump and treat.

Moreover, we use injectable filters in conjunction with lauric acid to simultaneously target heavy metal contamination (e.g., Cu^2+^) and hydrocarbons immiscible in water. Specifically, we inject lauric acid in canola oil in model polluted aquifers, to induce Cu^2+^ migration in toluene (used as model hydrocarbon), enabling its retention by injectable HEC + filters. Toluene and Cu^2+^ could then be removed more easily from groundwater, e.g., by pumping them in filter proximity, upstream of the filters. Lauric acid^73^ and oleic acid^74^ were previously used to extract Cu^2+^ from water, but their use was not combined with injectable barriers. Also, our previous research showed that the benign surfactant SLL also induced Cu^2+^ partitioning into hydrophobic solvents such as canola oil, used as liquid sorbent in the context of water purification^20^. However, in this case, the oil phase became solid and could not be pumped from the subsurface. Therefore, SLL was exclusively intended for ex situ water treatment following pump and treat^20^.

## Materials and methods

### Materials

CuCl_2_·2H_2_O and CuSO_4_·5H_2_O (ACS grade), lauric acid (ACS grade), toluene (HPLC grade), NaOH (pellets, ACS grade) and cationic hydroxyethyl cellulose (HEC +) were purchased from Sigma Aldrich (Canada). Canola oil (Selection brand) was purchased from a local market. THF (Caledon Laboratories Ltd, ACS grade), dioxane and isopropanol (Fisher Scientific Canada, ACS grade) were purchased from Fisher Scientific (Canada). Sand (EMD Millipore, Canada) was purchased from VWR. Deionised (DI) water was used in all experiments.

### Bottle tests

Bottle tests were conducted in glass vials, to investigate separation between THF and water, using 30%, 50% and 70% THF relative to water (v/v) and lauric acid concentrations ranging from 0.2 to 1 M, at pH = 2, 6.5 and 13. Equation (1) was used to estimate the difference between the volume of water added to the mixture (V_water,used_) and the volume of the water-rich bottom phase separated using lauric acid (V_water,measured_)

1 $$ \left( {{\text{V}}_{{{\text{water}},{\text{measured}}}} - {\text{V}}_{{{\text{water}},{\text{used}}}} /{\text{V}}_{{{\text{water}},{\text{used}}}} } \right)*{1}00\left( \% \right) $$

In the “Results and discussion” section this difference is referred to as deviation.

Bottle tests were also conducted to assess the partitioning of Cu^2+^ ions between the water and oil phases, and to study emulsification between toluene and water. Bottle tests were conducted by mixing first 5 mL of 30 M aqueous CuCl_2_ or CuSO_4_ solutions, 1 mL of 0.23 M lauric in canola oil solutions and either 1 mL or 4 mL of toluene. Afterwards, 10 mM NaOH (relative to the water phase) were added, and vials were agitated by hand for 30 s, before equilibrating them on a bench. Experiments were also conducted with pure water (without copper salts), using 1 mL of 0.23 M lauric in canola oil solutions and 1 mL of toluene, without NaOH or with 10 mM NaOH. Finally, additional bottle tests were conducted using 30 mM CuCl_2_ or CuSO_4_ solutions, toluene, and lauric acid alone, at pH = 13. The volume ratio of the aqueous phase to the toluene phase was four parts of water and one of toluene. All bottle tests were done in duplicate. Duplicates closely resembled each other.

### Static interfacial tension measurement

Static interfacial tension measurements were conducted using a Sigma force tensiometer (Biolin Scientific, USA) and a platinum du Noüy ring (having a 2 cm diameter), using four types of samples, the composition of which is summarized in Table 1. The water and oil phases were mixed (1:1.2 water:oil, v/v) and allowed to separate in a separatory funnel for 24 h. They were then collected separately and carefully transferred in a glass cup with the aid of a pipette, to measure interfacial tension.

**Table 1:** Samples used for static interfacial tension measurements.

Sample	Water phase	Oil phase
1	water	2:1 toluene: 0.23 M lauric acid in canola oil solution (v/v)
2	water + 10 mM NaOH	
3	30 mM CuCl_2_ + 10 mM NaOH	
4	30 mM CuSO_4_ + 10 mM NaOH	

The surface tension provided in the “Results and discussion” section was estimated using (2):

2 $$\gamma =\frac{F}{4\pi R}f$$

where F is the maximum force measured when pulling the du Noüy ring outside of the water phase (i.e., the force required to break the lamella), R is the average radius of the Du Nuoy ring used and *f* is the Huh and Mason correction factor calculated as f = R/r (R = radius of the du Noüy ring and r = radius of the wire). All measurements were conducted in triplicate, to estimate the low standard deviation reported in the “Results and discussion” section.

### Compression isotherms

Compression isotherms were measured at the oil–water interface using a Kibron Microtrough G1 Langmuir–Blodgett trough (Kibron, Sweden), controlled using KBN LayerXPro software (Kibron, Sweden). The water and oil phases are as described for samples 3 and 4 used for static interfacial tension measurements (Table 1, “Static interfacial tension measurement”). Samples were prepared in a separatory funnel, agitated and allowed to separate overnight. The water and oil phases were then collected separately, and carefully transferred into the lower and upper compartment of the Langmuir trough, respectively (with the aid of a pipette). After transferring the top and bottom phases, interfacially active species were allowed to adsorb at the interface for 10 min, before being compressed from 16,500 to 1650 mm^2^, using mobile barriers moving at a speed of 20 mm/min. After the first compression the barriers were rapidly expanded (at a speed of 140 mm/min) and films were immediately recompressed at 20 mm/min. The pressure was monitored using a Wilhelmy plate during each compression. Measurements were conducted in duplicate and measured curves closely resembled each other.

### ATR-FTIR

ATR-FTIR measurments were conducted to investigate potential interactions between lauric acid and Cu^2+^ ions. In these measurments, the samples used were prepared as described in “Bottle tests”. Specifically, we mixed first 5 mL of 30 M aqueous CuCl_2_ or CuSO_4_ solutions, 1 mL of 0.23 M lauric acid in canola oil solutions and 1 mL of toluene. Afterwards, 10 mM NaOH (relative to the water phase) were added, and vials were agitated by hand for 30 s, before allowing to settle on the bench. Experiments were also conducted without copper salts, using 1 mL of 0.23 M lauric in canola oil solutions and 1 mL of toluene, with 10 mM NaOH. Only the oil phase was analyzed, after 24 h equilibration. Additionally, ATR-FTIR was used to analyze samples prepared using 3:7 THF:water mixtures (v/v), using either water or 0.03 M CuCl_2_ or CuSO_4_ aqueous solutions. Absorbance spectra were collected using an ATR-FTIR spectrometer (Thermoscientific Nicolet Summit FTIR spectrometer with an Everest ATR), with an accompanying IR solution software. Due to the volatility of THF, each spectrum is the average of only 10 scans, with a resolution of 4 cm^−1^, in the wavenumber range of 400–4000 cm^−1^. Measurements were repeated at least four times per sample to account for the low scan count.

The absorbance peak associated with the OH stretch (H-bonding) of 3:7 v/v THF:water mixtures (pH ≈ 6.5 and pH ≈ 3) separated with 1 M lauric acid was deconvolved using a coarse-grain approach developed by us, as described in our previous manuscripts^75,76^. Briefly, normalized experimental data [0–1] in the range 2500–4000 cm^−1^ were fitted to a sum of two Gaussians by nonlinear regression using Graphpad Prism 9.2.0 (Eq. 3). This software uses the Levenberg and Marquardt algorithm^77,78^. We excluded the C–H stretching intensities in the range 2650–3000 cm^−1^^79^, to improve the stability of the fit considerably and remove non-water OH contributions from the analysis.

3 $$ Intensity = A_{1} exp^{{ - 0.5\,\;\left( {\frac{{(x - \nu_{1} )}}{{SD_{1} }}} \right)^{2} }} + A_{2} exp^{{ - 0.5\,\;\left( {\frac{{(x - \nu_{2} )}}{{SD_{2} }}} \right)^{2} }} $$

We did not use any weighting for a maximum of 1000 iterations with medium convergence criteria. The three replicate Y values collected from three independent spectra were considered an individual point. All parameters were constrained to be greater than zero, and symmetrical confidence intervals and standard errors were calculated. All r^2^ values for the fits were greater than 0.9989.

Our approach was based on the simulations of water structure reported by Lenz and Ojamäe (2006)^80^. These authors partitioned the vibrational spectrum into contributions from different molecules according to their coordination properties, e.g., double H-bond (DD) or single H-bond (SD) donors. In our coarse-grained approach, shifts to higher wavenumbers (blueshifts) would indicate an enhancement in the structure of water.

All other spectral regions were analyzed using Omnic 9 (Thermo Fisher Scientific). Spectra were baseline corrected using independent spline baselines. Peak areas were determined either using Omnic 9 (for entire regions) or using Peakfit v4.12 (Seasolve) for individual peaks.

### Optical microscopy

An optical VHX-5000 digital microscope (Keyence Corporation, Canada) was used to image samples described in “Bottle tests”. Each type of sample was imaged at least 10 times, to ensure that images were representative of the real sample characteristics.

### Flow experiments through injectable filters

Injectable filters were obtained as described elsewhere^51,81^. Briefly, a glass graduated cylinder having a 14 mm diameter was perforated at the bottom and filled with sand for a total volume of 5 mL, to mimic an aquifer. Water (20 mL) was flushed through the cylinder before injecting 1 mL of 0.1% wt HEC + solutions in water, followed by rinsing with excess water (60 mL). We subsequently injected in the cylinder co-contaminant mixtures of copper salts and toluene (samples 3 and 4 described in Table 1, “Static interfacial tension measurement”). Note that samples filtered through injectable filters were not allowed to settle overnight. The eluent was collected from the bottom of the cylinder and analyzed using ICP-OES (as described in “ICP-OES”), to assess copper concentrations in the water phase. Experiments were conducted in duplicate.

### ICP-OES

ICP-OES tests were conducted in triplicate using a PerkinElmer 5300 DV instrument, to quantitate the removal of copper ions from CuCl_2_ and CuSO_4_ aqueous solutions, using lauric acid. The summary of the samples analyzed is provided in Table 2. Bottle test samples were allowed to settle overnight, before collecting the water phase. The eluent from flow experiments described in “Flow experiments through injectable filters” was not allowed to settle overnight (i.e., samples were filtered immediately after mixing and the eluent was immediately collected). Each measurement was conducted in triplicate.

**Table 2:** Samples used for ICP-OES tests.

Water phase composition (5 mL)	Oil phase (total volume in brackets)
**Bottle test samples**	
30 mM CuCl_2_ or CuSO_4_ + 10 mM NaOH	1:1 toluene: 0.23 M lauric in canola oil solution (2 mL)
10 mM CuCl_2_ or CuSO_4_ + 10 mM NaOH	1:1 toluene: 0.23 M lauric in canola oil solution (2 mL)
10 mM CuCl_2_ or CuSO_4_ + 10 mM NaOH	1:1 toluene: 0.08 M lauric in canola oil solution (2 mL)
30 mM CuCl_2_ or CuSO_4_ + 10 mM NaOH	1:4 toluene: 0.23 M lauric in canola oil solution (5 mL)

Water phase (5 mL)	Oil phase (volume in brackets)
**Flow tests/filtration experiments**	
30 mM CuCl_2_ or CuSO_4_ + 10 mM NaOH	1:1 toluene: 0.23 M lauric in canola oil solution (2 mL)

### Synchrotron based Small Angle X-ray Scattering (SAXS)

SAXS experiments were carried out at the Canadian Light Source Synchrotron (CLS) on the Brockhouse Diffraction Sector Undulator Beamline (BXDS-IVU)^82^, to identify the self-assembly of lauric acid (500 g/L) into crystal structures into pure THF and in THF-water mixtures. Samples were prepared using water at circum-neutral and acidic pH (pH = 2) and with different THF-water ratios (90–95% THF relative to water, v/v). Samples were pipetted and sealed in a Kapton tubing (Polyamide 0.0575″ ID × 0.0615″ OD) with wax to prevent solvent evaporation during data collection. SAXS data was collected with a Rayonix MX300HE detector (8192 × 8192 pixels) with 2 × 2 binning (4096 × 4096 pixel) for an effective pixel size of 73.242 µm with background stability mode ON. SAXS patterns were collected with a photon energy of 12.18 keV and sample-to-detector distance of ~ 233 cm. SAXS patterns were collected in transmission geometry with a 10 s dwell time. Patterns were processed with GSASII (Argonne National Laboratory (C), 2010) This product includes software developed by the UChicago Argonne, LLC)^83,84^. SAXS data was calibrated with silver behenate (AgBeh) and instrument parameters such as sample-to-detector distances, detector tilt, beam centre, were refined as described elsewhere^83^. SAXS patterns were integrated from q = 0.012 to q = 0.637 Å^−1^. Measurements were duplicated for each sample.

The scattering intensity was normalized [0,1] and fitted by nonlinear regression with Graphpad Prism 9.4.0 (Graphpad software, San Diego, California). Prism uses the Levenberg and Marquardt algorithm for non linear regression^77,78^. We fitted three different models to the data: Gaussian, Teubner and Strey, and Ornstein–Zernike.

The Gaussian model is given by Eq. (4).

4 $$I\left(q\right)=A\cdot \mathrm{exp}\left(-\frac{1}{2}{\left(\frac{x-\mu }{\sigma }\right)}^{2}\right)$$

where I(q) is the scattering intensity (expressed as a function of the scattering vector q), A is the amplitude (A = 1 in this case, since the data were normalized), σ is the standard deviation, and μ is the mean.

The Teubner and Strey model^85^, shown in Eq. (5), was fitted to the normalized scattering intensity vs. q data.

5 $$I\left(q\right)=\frac{C}{{a}^{2}+{c}_{1}{q}^{2}+{c}_{2}{q}^{4}}$$

where C is a proportionality constant, set to 1 in our treatment due to data normalization, and a, c_1_ and c_2_ are fitting parameters, which were used to determine the periodicity d and the correlation length ξ, as shown in Eqs. (6)–(7).

6 $$\xi ={\left[\frac{1}{2}{\left(\frac{{a}_{2}}{{c}_{2}}\right)}^{1/2}+\frac{1}{4}\frac{{c}_{1}}{{c}_{2}}\right]}^{-1/2}$$

7 $$d={2\pi \left[\frac{1}{2}{\left(\frac{{a}_{2}}{{c}_{2}}\right)}^{1/2}-\frac{1}{4}\frac{{c}_{1}}{{c}_{2}}\right]}^{-1/2}$$

The Ornstein–Zernike model was fitted to normalized scattering intensity vs. q data using Eq. (8).

8 $$I\left(q\right)=\frac{1}{{a}^{2}+{c}_{1}{q}^{2}}$$

### ^1^H NMR

^1^H NMR was used to analyze the purity of the water-rich phase separated from samples prepared with 0.125 M or 1 M lauric acid and mixtures of 7:3 water:THF (v/v). Samples were transferred directly into 5 mm NMR tubes without the addition of any deuterated solvent. NMR spectra were collected on a Bruker AVANCE NEO 300 MHz spectrometer equipped with a 5 mm BBFO probe. The sample temperature was regulated at 298 ± 1 K. Quantitative spectra were collected with the field frequency lock turned off, using a single scan with a relaxation delay of 30 s and an acquisition time of 5.6 s. Spectra were processed with 0.3 Hz line broadening and polynomial baseline correction.

Under quantitative NMR conditions, the integral of an NMR peak is proportional to the number of protons contributing to the peak (e.g., two for H_2_O and four for THF). Thus, in a two-component THF-water system, the mole fraction of one component (e.g., water in the water-rich phase) is given by Eq. (9)

9 $${\chi }_{water}=\frac{\frac{{I}_{water}}{2}}{\frac{{I}_{water}}{2}+\frac{{I}_{S}}{4}}$$

where ${I}_{water}$ and ${I}_{S}$ are the NMR peak integrals of water and either THF, respectively. Note that the integration limits for the organic solvents encompass both the main peak and the ^13^C satellite peaks.

The above equation is only true for a two-component system. While the NMR spectra did reveal the presence of lauric acid in the sample, the mole fraction of lauric acid was less than 0.0003 in the water-rich fraction of all samples. As such, the water-rich phase of all samples is effectively a two-component system for the purpose of this analysis.

The partial molar volumes of THF and water^86^ were used to compute %v/v water from ${\chi }_{water}$.

Each sample was analyzed in triplicate.

### Light scattering

Light scattering experiments were conducted in triplicate at room temperature (23 °C) using a Malvern Zetasizer Nano ZSP. Samples were equilibrated for 120 s prior to each measurement and measurements were repeated at least three times for every sample. The refractive index used was 1.3 for water^18,87^ and 1.423 for lauric acid. The density, viscosity and refractive indexes of THF-water mixtures depended on the THF:water ratio, and are as reported elsewhere^88,89^. Measurements were conducted in triplicate.

## Results and discussion

This section discusses emulsification between toluene and water, and separation between water and THF in the presence of lauric acid (“Emulsification and solvent separation by lauric acid (without Cu2+)”). We also discuss the mechanisms through which copper ions migrate in the organic phase following solvent separation, in the presence of lauric acid (“Partitioning of Cu2+ ions between the organic solvent and the water phase”). We analyze how copper ions decrease the stability of water-toluene and THF-water emulsions, promoting the separation between free water-rich and organic solvent-rich phases, where copper ions partition (“Emulsion stability with copper salts”). Finally, “Flow test experiments (injectable filters)” discusses injectable filters, which can be used in conjunction with lauric acid for the treatment of copper and toluene pollution in groundwater.

### Emulsification and solvent separation by lauric acid (without Cu^2+^)

Lauric acid emulsifies toluene and water, and emulsion stability increases from pH = 2 to pH = 6.5, and from pH = 6.5 to pH = 13 (Fig. S1, supporting information file). Emulsion stability at basic pH is correlated to low interfacial tension. The interfacial tension measured at the toluene-water interface, using 0.125 M lauric acid (relative to the toluene phase) is similar at acidic pH (20.0 ± 1.1 mN/m) and at circum-neutral pH (24.2 ± 2.7 mN/m), and lower at basic pH (13.0 ± 0.4 mN/m).

The pKa of lauric acid is ≈5^90^. A study conducted in pure water reports that at pH < 4, lauric acid is not dissociated, at circum-neutral pH a portion of lauric acid is in the form of laurate ions, and at pH > 9 all of lauric acid in aqueous solution is in the form of laurate ions^91^. While lauric acid is insoluble in water, laurate ions have greater affinity for the water phase^92^. Interfacial tension measurements show that laurate ions are more interfacially active and more effective than lauric acid at stabilizing toluene-water emulsions.

Other studies conducted using lauric acid, water and water-immiscible oils also report that the pH of the water phase affects emulsification. For instance, a study conducted using solutions of lauric acid in paraffin and mineral oil reports that sodium laurate films form at the oil–water interface with NaOH > 0.2 M^93^. These films stabilize droplets because of their negative electrostatic charge at alkaline pH^93^. Another study reports that mixed films of lauric acid and laurate enhance the stability of water-cyclohexane and water-n-hexadecane emulsions at basic pH^94^. Moreover, the oil:water ratio affects emulsion characteristics. As an example, a previous study reports that at high water concentrations, micelles incorporate oil in the aqueous surfactant solution, whereas at high oil:water ratios micelles incorporate water in oil^93^. Here, the toluene:water ratio used (7:3 toluene:water, v/v) enables the formation of both oil in water and water in oil emulsions at basic pH, as evident from the turbidity of both the toluene and the water layers.

**Figure 1:**
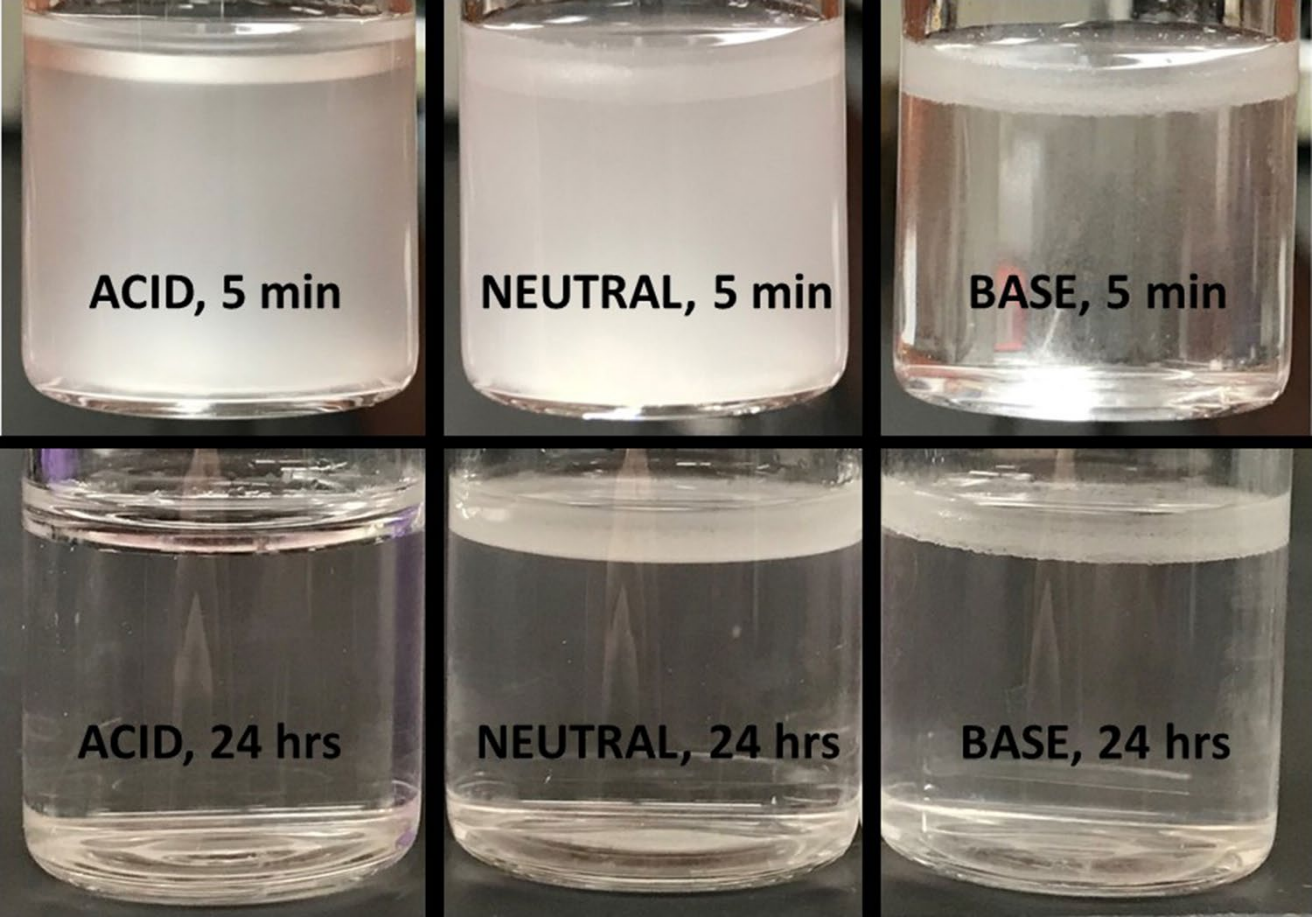
Separation between THF and water with 0.125 M lauric acidic, at basic, acidic and circum-neutral pH after 24 h (bottom row) and after 5 min (top row). The THF-water mixture contains 30% THF relative to water, v/v. Images collected 5 min after mixing with 1 M lauric acid are in the supporting information (Fig. S2).

**Figure 2:**
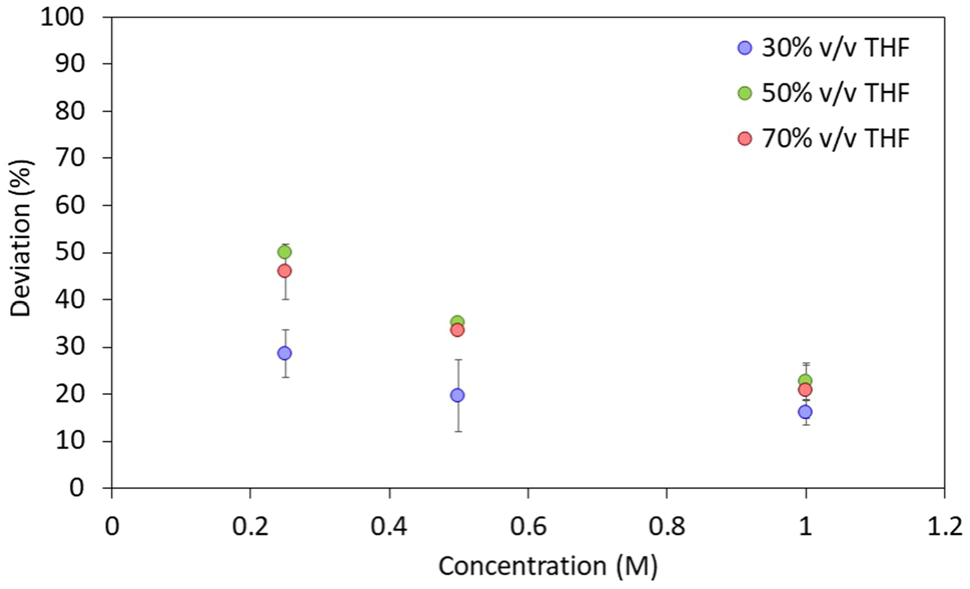
Separation between THF and water at circum-neutral pH (pH = 6.5), with different lauric acid concentrations ranging from 0.2 to 1 M (as indicated on the x axis). Volumes were measured after centrifuging samples for 10 min at 3500 RPM. The THF percentages reported in the legend are relative to the water phase (v/v). The deviation on the ordinate axis was measured using Eq. (1) as follows (V_water,measured_-V_water,used_/V_water,used_)*100(%).

In addition to emulsifying toluene and water, lauric acid also separates THF from water (Fig. 1 and Fig. S2, supporting information file). The purity of the THF and water phases is given in Table 3 (as determined by ^1^H NMR). The data show that the pH has negligible effect on the purity of the separated water phase after 24 h equilibration. In contrast, lauric acid concentration affect water purity. At circum-neutral pH, purity of the water-rich phase increases with increasing lauric acid concentration (from 0.125 to 1 M, Fig. 2). Nonetheless, using excessively high lauric acid concentrations would not be feasible for practical purposes, and we therefore focus on lower concentrations (e.g., 0.125 M).

**Table 3:** ^1^H NMR analysis of the water-rich separated layer, obtained starting from 30% v/v THF-70% v/v DI water mixtures, and either 0.125 M or 1 M lauric acid.

Sample pH	THF in water layer (% v/v)	water in water layer (% v/v)
Acidic, 0.125 M	24.2 ± 0.4	75.8 ± 0.4
Acidic, 1 M	13.4 ± 0.6	86.6 ± 0.6
Circum-neutral, 0.125 M	24.2 ± 0.4	75.8 ± 0.4
Circum-neutral, 1 M	13.6 ± 0.2	86.4 ± 0.2
Basic, 0.125 M	24.5 ± 0.5	75.5 ± 0.2
Basic, 1 M	14.0 ± 0.1	86.0 ± 0.1

Samples were centrifuged before analyzing each layer.

**Figure 3:**
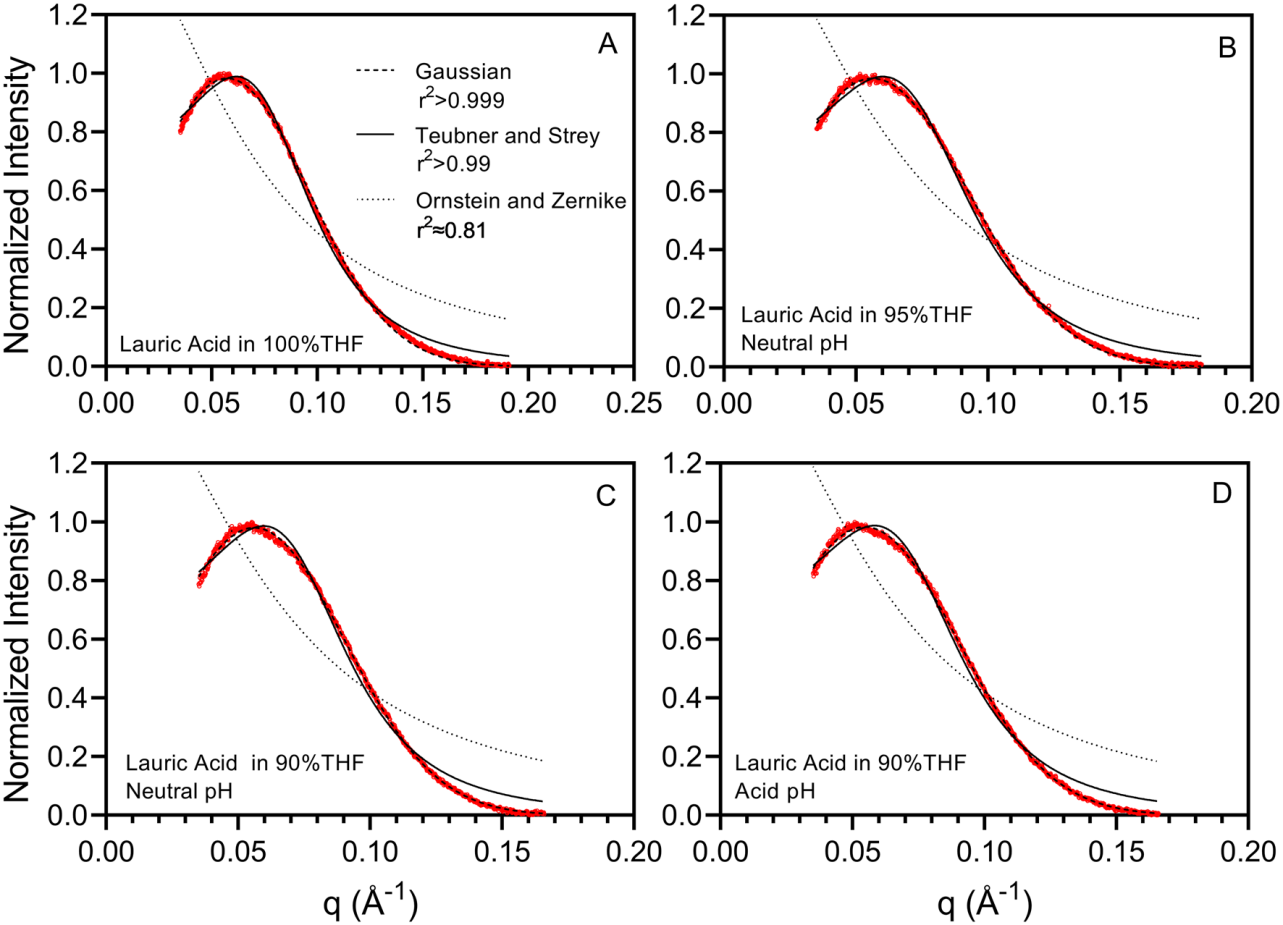
SAXS patterns of lauric acid in either 100% THF, or in THF-water mixtures, with 95% THF or 90% THF (at either acidic or circum-neutral pH) relative to water (v/v) and 500 g/L lauric acid. The experimental data are shown in red, and fit to the Steubner and Strey model (continuous black line) or a Gaussian (dashed line). The Ornstein and Zernicke fit (dotted line) is poor, and shown as a reference. The best fitting parameters of the Ornstein and Zernicke model are as follows: 100% THF: a_2_ = 0.6572, c_1_ = 152.8; 95% THF, neutral pH: a_2_ = 0.6366, c_1_ = 167.0; 90% THF, neutral pH: a_2_ = 0.6402, c_1_ = 173.0; 90% THF, acidic pH: a_2_ = 0.6249, c_1_ = 175.9. The Teubner and Strey best fitting parameters are as indicated in Table 4.

We hypothesize that THF-water separation starts with water partitioning inside self-assembled lauric acid structures. SAXS measurements were conducted to characterize these structures, using THF and THF-water mixtures (containing 90% and 95% THF relative to water, v/v, at circum-neutral and acidic pH, Fig. 3). SAXS patterns are characterized by a single peak, which was fit using the model developed by Teubner and Strey for reverse micelles, which are a thermodynamically stable fluid comprised of water, a hydrophobic solvent, and an amphiphile^85^. This is different from a kinetically stable emulsion, which separates into a hydrophobic solvent phase and a water phase over time. Samples used in SAXS measurements had a water- to-surfactant molar ratio of 2.2 or less.

In the Teubner and Strey model, the intensity is described by Eq. (2), as reported in the “Materials and methods” section. For the purpose of the fit, in our study the $\sim$ sign in the original expression by Teubner and Strey^85^ was substituted with an equal sign in Eq. (2). Note that in Eq. (2) we set the proportionality constant C = 1, because normalized data were used.

The Teubner and Strey model is derived from the phenomenological model developed by Landau to describe changes in free energy *F*(*η*) during phase transitions (where *η* is the order parameter). When a system is not undergoing transitions, *η* is constant. The free energy *F*(*η*) is obtained as the integral of the free energy density *f*(*η*). Near the phase transition, changes in *η* are small and *f*(*η*) can therefore be approximated by a truncated Taylor expansion in *η*^95^. The coefficients of the Taylor expansion are the fitting parameters which appear in Eq. (2). The order at which the expansion is truncated depends on the problem analyzed and the accuracy desired^85^. Note the difference between the Teubner and Strey model (which accounts for terms up to q^4^) and the Ornstein–Zernike model (Eq. 8), which is obtained when considering only the components corresponding to small order parameter fluctuations and only accounts for terms up to q^2^.

The Ornstein–Zernike equation was developed by Leonard Ornstein and Frits Zernike^96^. The Ornstein–Zernike equation has been used to estimate the structure factor of liquids and colloids in scattering experiments. While it has been used to study liquids, it is not suitable to describe reverse micelles, as reported by Teubner and Strey^85^ and as shown in Fig. 3.

The periodicity d and the correlation length ξ for the Teubner and Strey model are provided in Table 4, alongside with the fitting parameters for the samples analyzed.

**Table 4:** Periodicity *d* and to the correlation length ξ estimated using the Teubner and Strey model for different samples containing lauric acid (500 g/L) in either THF (100% THF) or THF-water mixtures (with 90% or 95% THF relative to water, v/v), at neutral and acidic pH.

	Fitting parameters	ξ (Å)	d (Å)	d/ξ
**100% THF**				
a_2_	1.401	24.3	84.8	3.48
c_1_	− 2.069·10^2^			
c_2_	27.258·10^3^			
**95% THF, neutral pH**				
a_2_	1.419	25.4	87.4	3.44
c_1_	− 2.272·10^2^			
c_2_	31.412·10^3^			
**90% THF, neutral pH**				
a_2_	1.461	26.4	89.0	3.36
c_1_	− 2.522·10^2^			
c_2_	35.469·10^3^			
**90% THF, acidic pH**				
a_2_	1.409	25.8	89.7	3.48
c_1_	− 2.335·10^2^			
c_2_	34.310·10^3^			

The fitting parameters for the samples are also provided. Note the negative values of c_1_, typical of reverse micelles^85^.

The good fit of the Teubner and Strey model to the experimental data indicates that lauric acid self-assembles into reverse micelles, which host water in their interior. Note that we also used this model to describe SAXS patterns measured in pure THF as solvent. While water was not added, the solvent was not dried, and water impurities would have been present. The correlation length ξ identified with the Teubner and Strey model in pure THF is the diameter of a reverse micelle, since it is approximately twice the size of a lauric acid monomer reported by an atomic simulation study^97^.

Based on the Teubner and Strey model, lauric acid self-assembles into micelles, the size of which increases with increasing water content, at neutral pH, as indicated by the increasing value of ξ. This result is attributed to the fact that as the water content increases, the amount of water partitioned into the reverse micelles also increases. With 90% THF and 10% water, the reverse micelle size is greater at neutral than at acidic pH, at which the hydrophilic heads of lauric acid would have a lower negative charge density, leading to decreased electrostatic repulsion and tighter packing. Previous atomistic simulation studies report that the self-assembly of lauric acid in pure water is pH-dependent^91,97^. At pH = 13 it self-assembles into micelles, whereas at pH = 4–9 it self-assembles into bilayers, and the order parameter increases with decreasing pH^91,97^. In our study, the solvent was either THF or a mixture of THF and water. Organic solvents in solution would induce the self-assembly of lauric acid into reverse micelles, as reported in a study which used it to remove methylene blue from water, by causing its migration into isopentanol (used as the organic solvent)^98^.

The periodicity *d* determined based on the Teubner and Strey model is ≈3.4 times the correlation length ξ, indicating that reverse micelles of lauric acid aggregate. The periodicity d is interpreted as the average distance between the centers of lauric acid reverse micelles within the aggregates. Aggregation between reverse micelles is ascribed to the high lauric acid concentrations used in SAXS measurements.

Note that while a Gaussian model provides a good mathematical fit to the data (Fig. 3), it does not adequately capture the physical characteristics of microemulsions. Based on a Gaussian model, the micelle size is estimated as 2π/q_max_ (where q_max_ is the position of the peak maximum) and is 109–115 Å for the different samples analyzed (Table S4, supporting information file). This size is larger than the one determined with Teubner and Strey, and does not correlate well to the size of lauric acid molecules.

Light scattering experiments were conducted to complement SAXS results, to probe different length scales. With 90% DMSO, droplet size was above the range of detection of the zeta-sizer used for light scattering experiments, and samples phase separated after 48 h at either circum-neutral or acidic pH. With 95% THF, light scattering experiments reveal that droplets are 450 ± 82 nm and 505 ± 176 nm at circum-neutral and acidic pH, respectively.

**Figure 4:**
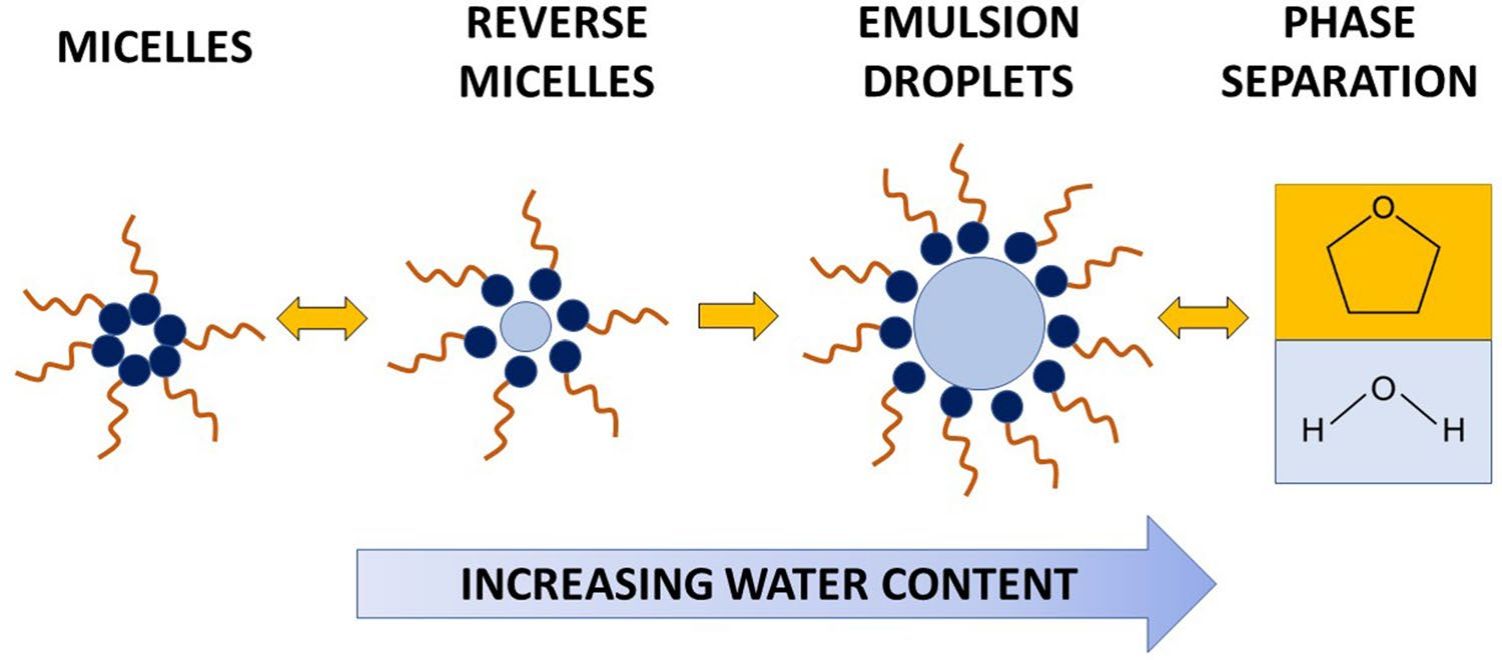
Proposed mechanism of THF-water separation by lauric acid. Note that this mechanism occurred when mixing lauric acid in its dry form to THF-water mixtures. Mixing dry lauric acid in water is an option when treating surface water. Delivery of lauric acid in polluted aquifers requires that its is dissolved in a liquid carrier, e.g., canola oil, as discussed later and schematized in Fig. 11.

In summary, based on our results we hypothesize that reverse micelles are the precursors to droplets, and that they grow upon increasing the water content in THF, in agreement with previous studies^99^. Reverse micelles would grow into droplets, and progressive increase in droplet size would ultimately lead to phase separation (Fig. 4). Recall that bottle tests show that at circum-neutral pH, emulsions are water in THF (rather than THF in water). Therefore, the data suggest that when the percentage of water exceeds the percentage of THF, reverse micelles swell to host increasing volumes of water in their interior and partition in the THF-rich phase.

ATR-FTIR was used to analyze intermolecular interactions between THF, water and lauric acid. This analysis was conducted to probe if lauric acid interfered with THF-water interactions, inducing solvent separation. THF and water interact through H-bonds^100^. Species such as sugars and sodium lauroyl lactylate^64^ preferentially interact with water through H-bonds, causing separation between water and THF or acetonitrile^76^. At circum-neutral pH, part of lauric acid should be in the form of laurate ions (as discussed earlier). Although laurate ions are interfacially active, analysis of the H-bond region indicates that lauric acid had a negligible effect on H-bonding of the water phase at either acidic or circum-neutral pH (Fig. S3 and Tables S1–S3, supporting information file).

Since water-THF separation by lauric acid cannot be explained based on the effect of lauric acid on H-bonding of water, further ATR-FTIR analyses were conducted on samples containing 1 M lauric acid in 3:7 THF:water (v/v), at circum-neutral, acidic and basic pH. Specifically, we analyzed the top and bottom phase of the samples, to determine the purity of the separated phases and to probe the effect of pH on lauric acid partitioning between phases. The spectra of all THF-water mixtures (without lauric acid) were largely similar at all pH values analyzed (Fig. S5, supporting information file). The spectra of the bottom water-rich phase were also spectroscopically identical at all pH values (Fig. S6, supporting information file). Moreover, the spectra of the bottom layers of THF-water mixtures separated by lauric and of THF-water mixtures without lauric acid are largely similar, with the exception of the intensity of the THF band in the 2900 cm^−1^ region (inset of Fig. S6, supporting information file). This result indicates that the water-rich layer separated by lauric acid does not contain lauric acid (i.e., lauric acid partitions mainly in the THF-rich phase). The intensity difference seen in the CH stretching region can be used to roughly estimate the THF content in the bottom water-rich layer. Integrating this peak and comparing its area to the one of the peak measured in 3:7 THF:water mixture (before separation with lauric acid), we determined that the THF content in the bottom layer is 18.5%, in agreement with ^1^H NMR data.

**Figure 5:**
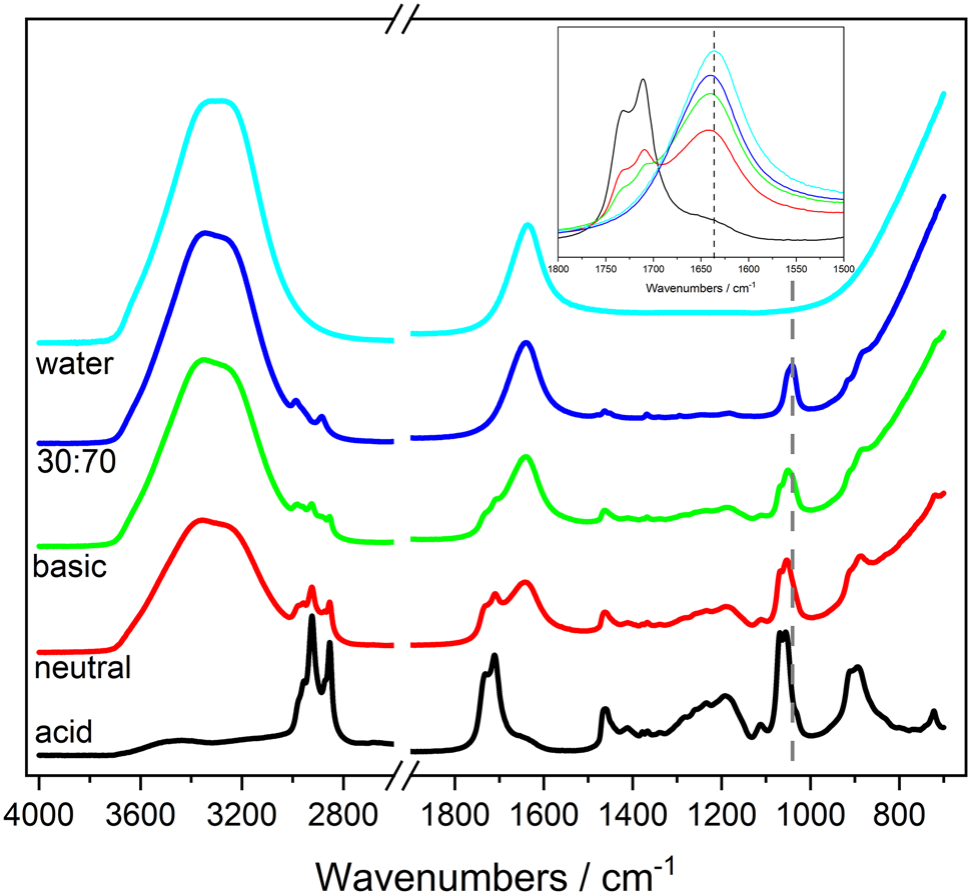
Spectra of the top (THF-rich) layer of THF-water mixtures separated using 1 M lauric acid under acidic (black), circum-neutral (red) and basic (green) conditions. THF-water mixtures were prepared using 30:70 THF:water (v/v) mixtures. The spectra of 30:70 THF:water (v/v) mixtures (without lauric acid) (blue) and the spectra of pure water (cyan) are also shown for comparison. The inset shows the carbonyl and water bending region of the spectra. The grey dashed line indicates the shift in the THF C–O stretch peak.

Figure 5 shows the spectra of 3:7 THF:water mixtures (without lauric acid) and of the top layer of mixtures separated by lauric acid, at different pH values. Tables 4 and 5 highlight relevant absorbance peaks. The spectrum of water is also included for comparison. The spectra of lauric acid powder, pure THF and 1.5 M lauric acid in THF are shown in Fig. S4 (supporting information file), as a reference. The broad band at wavenumbers greater than 3000 cm^−1^ corresponds to O–H stretching vibrations^76^. The peaks between 3000 and 2800 cm^−1^ are due to the CH stretching vibrations of both THF and lauric acid^101^. The double peak centred at 1720 cm^−1^ corresponds to the C = O stretching mode of lauric acid, specifically to lauric acid molecules with two hydrogen bonds (low frequency) and with one hydrogen bond (high frequency)^102^. The water bending mode (δ_HOH_) is at 1640 cm^−1^^103^. At lower frequencies, the fingerprint region contains a number of peaks corresponding to characteristic vibrations, including aliphatic bending (at 1460 cm^−1^,^104^) and C–O stretching (at ~ 1060 cm^−1^,^105^) modes. Note that the spectra do not show the characteristic peaks of carboxylates around 1580–1550 cm^−1^ (for the antisymmetric COO^−^ vibrations) and 1417–1308 cm^−1^ (for the symmetric COO^−^ vibrations). This indicates that the top layer emulsion contains lauric acid in its protonated form only. Although the spectra of the bottom water-rich layer are identical for all pH values, the intensity of the top-layer carbonyl stretching band at 1720 cm^−1^ decreases with decreasing pH. Therefore, we hypothesize that the deprotonated form of lauric acid preferentially partitions at the interface between the water-rich and the THF-rich layers. This hypothesis is further supported by our interfacial tension measurements, as will be discussed later in this section.

**Table 5:** Frequency of the water bending mode (δ_HOH_) for each sample, prepared either with or without 1 M lauric acid.

Sample	δ_HOH_ frequency/cm^−1^
Pure water	1636
Top layer—basic	1641
Top layer—circum-neutral	1643
Top layer—acidic	1654^a^
3:7 THF:water (v/v) mixture (without lauric acid)	1640

In the table, the rows labelled as ‘top layer’ refer to the top layer of 3:7 THF:water (v/v) mixtures separated with 1 M lauric acid.

^a^Determined through second derivative analysis.

Wu et al. used the shift of the water bending band 1640 cm^−1^ (inset, Fig. 5) to quantify the water content in mixtures of water and glycerol, with higher frequencies corresponding to lower water content^106^. Table 5 shows the δ_HOH_ frequency for each sample. These values indicate that the water content increases with pH and in all cases is lower than in the 3:7 THF:water (v/v) mixture (without lauric acid).

This observation is further supported by analysis of the O–H stretching region. Indeed, the area under the O–H stretching band increases with increasing pH, indicating that more water is present in the THF phase at higher pH. However, the shape of the band is identical for all pH values, indicating that the nature of H-bonds is the same at all pH values. Hence, differences between separation efficiency and emulsion stability are not due to changes in H-bonding in the bulk THF and water phases.

The C–O stretch peak of THF for each of the samples also undergoes a shift, as indicated by the grey dashed line in Fig. 5. The higher frequency shoulder centred at 1068 cm^−1^ corresponds to the C–O stretch from the carboxylic group of lauric acid, whereas the lower frequency peak ranging from 1055 to 1040 cm^−1^ corresponds to the C–O stretch of the THF. Purkayastha and Madhurima show the spectra of progressively hydrated THF, where the C–O stretch band shifts to lower frequencies as a function of hydration^107^. In our case, the C–O stretch band shifts from 1066 cm^−1^ for pure THF to 1040 cm^−1^ for 3:7 THF:water mixtures without lauric acid. The shift seen in the absorbance peaks of ATR-FTIR spectra is summarized in Table 6. The data indicate that that the top layer of separated THF-water mixtures is enriched in THF compared to 3:7 THF:water mixtures (without lauric acid), at all pH values. Also, the molar fraction of THF in the top layer is pH-dependent. It is highest at acidic pH, at which the analysis of the water bending peak indicates that the water content of THF is lowest. This result is in agreement with bottle tests, which show that water in THF emulsions are least stable at acidic pH.

**Table 6:** Frequency of the C–O stretch of THF for each sample, prepared either with or without 1 M lauric acid.

Sample	Frequency C–O stretch/cm^−1^
Pure THF	1066
Top layer—acidic	1056
Top layer—circum-neutral	1053
Top layer—basic	1050
3:7 THF:water mixture (without lauric acid)	1041

In the table, the rows labelled as ‘top layer’ refer to the top layer of 3:7 THF:water (v/v) mixtures separated with 1 M lauric acid.

With 1 M lauric acid, the interfacial tension between THF and water is 2.96 ± 2.56 mN/m at pH = 2, 4.46 ± 0.02 mN/m at circum-neutral pH, and 9.51 ± 0.01 mN/m at pH = 13. In the case of immiscible fluids, the adsorption of interfacially active species at the liquid–liquid interface decreases the interfacial tension. In the case of toluene-water, interfacial tension is lowest at the highest pH, as discussed earlier. Here, we speculate that changes in the interfacial tension between THF and water are associated both to the effect of lauric acid on the structure of the bulk solvents, and on the adsorption of laurate ions at the THF-water interface. At acidic pH, ATR-FTIR data show that undissociated lauric acid is present in THF much more so than at any other pH and likely does not adsorb at the THF-water interface to produce elastic films. (Once again, note the difference between THF-water and toluene-water samples. In the case of toluene, interfacial tension between toluene and water is highest at acidic pH, because undissociated lauric acid has limited interfacial activity). In contrast, at basic pH, ATR-FTIR data show that laurate ions are not present in either the top or bottom layers, although the amount of lauric acid in the top phase is at its lowest. We contend that laurate (carboxylate) ions partition at the THF-water interface. Adsorption of the carboxylate ions at the THF-water interface would yield stable interfacial films. With such films, the liquid lamella breaks when subjected to greater tension (compared to acidic pH), when moving the du Noüy ring from the water to the THF phase. In other words, the higher interfacial tension measured at basic pH than at acidic pH is ascribed to the elasticity of laurate films at the THF-water interface. At basic pH, we speculate that the elasticity and electrostatic charge of interfacial films of laurate ions explain THF-water emulsion stability^108^. Interfacial film thickness can also contribute to emulsion stability^109^, although we have not investigated it in this study.

Note that while this study focuses on THF-water separation, lauric acid also separates water from other water-miscible solvents. For instance, lauric acid separates isopropyl alcohol (IPA) from water, although higher concentrations are required (Fig. S7). Lauric acid also separates dioxane from water, although separation is poor (Fig. S8), likely due to the poorer solubility of lauric acid in dioxane. Dioxane is used to stabilize chlorinated compound formulations, and it poses environmental concerns because of its toxicity and mobility in groundwater^110,111^.

### Partitioning of Cu^2+^ ions between the organic solvent and the water phase

**Figure 6:**
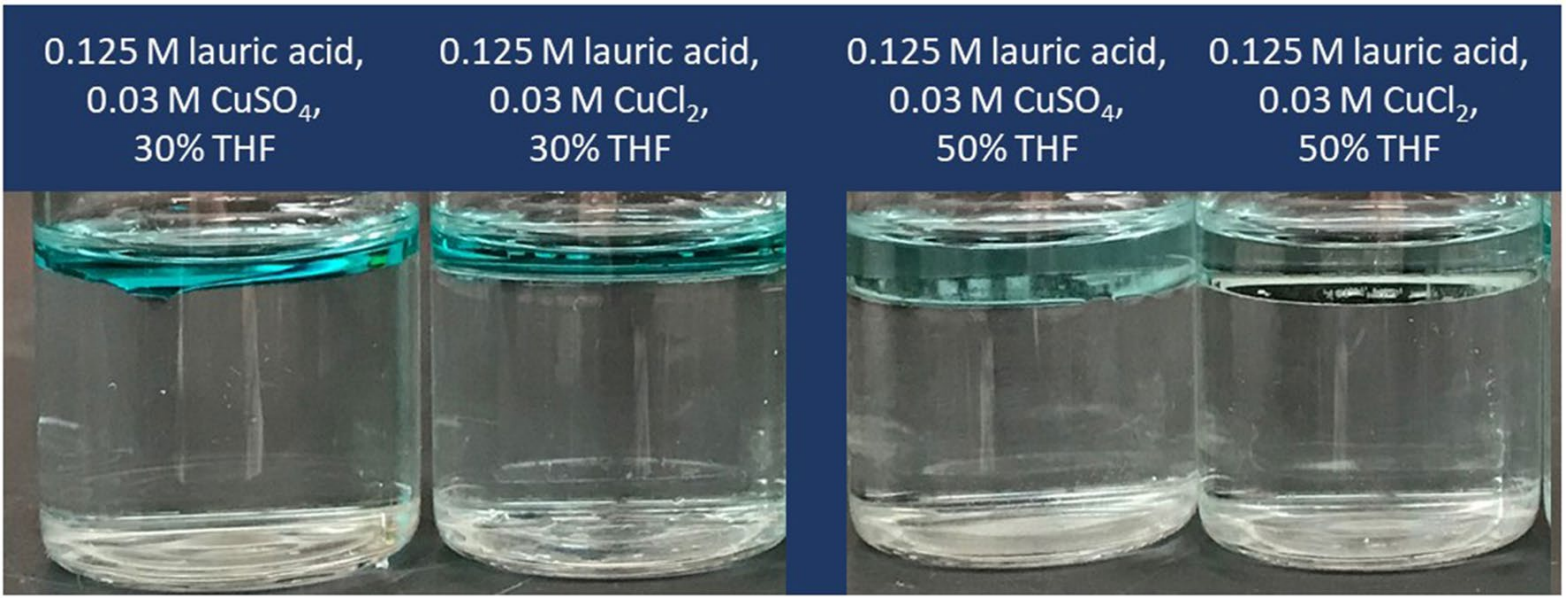
Bottle tests conducted using aqueous solutions of 0.03 M CuCl_2_ and CuSO_4_, using 30% or 50% THF v/v relative to water, at circum-neutral pH. Copper ions partition in the THF phase only at circum-neutral, while they remain solubilized in water at pH = 2 and precipitate in part out of solution at pH = 13 (Fig. S10, supporting information file). Images were taken after leaving vials to sit overnight.

As discussed in the previous section, lauric acid separates THF and water. Separation between THF and water also occurs with aqueous solutions of either 0.03 M CuSO_4_ or 0.03 M CuCl_2_ (Fig. 6). With 30% THF (relative to the water phase, v/v), 0.125 M lauric acid and at circum-neutral pH, the percent difference (deviation) between the initial water added and the volume of the water-rich layer after separation is 28.1 ± 1.0% and 28.1 ± 1.0% with 0.03 M CuCl_2_ and 0.03 M CuSO_4_, respectively. Increasing lauric acid concentration to 0.25 M decreases the deviation to approximately 25% with aqueous solutions of either 0.03 M CuSO_4_ or 0.03 M CuCl_2_. This result is similar to observations without copper salts (Fig. 2), which show increased THF-water separation with increasing lauric acid concentrations.

Upon THF-water separation, Cu^2+^ ions partition in the THF phase at circum-neutral pH, as qualitatively observed based on the discoloration of the top THF-rich layer (Fig. 6). This does not occur at pH = 2 (at which Cu^2+^ remains dissolved in water, Fig. S9, supporting information file). At pH = 13, part of the copper ions partition in the THF layer, and part precipitate out of solution, entraining laurate ions (as evident from the white color of the precipitates, Fig. S10, supporting information file). Note that precipitation of Cu^2+^ ions at basic pH would also occur without lauric acid. These results show that circum-neutral pH is preferable to achieve the simultaneous separation of THF and copper ions from water, when using lauric acid.

**Figure 7:**
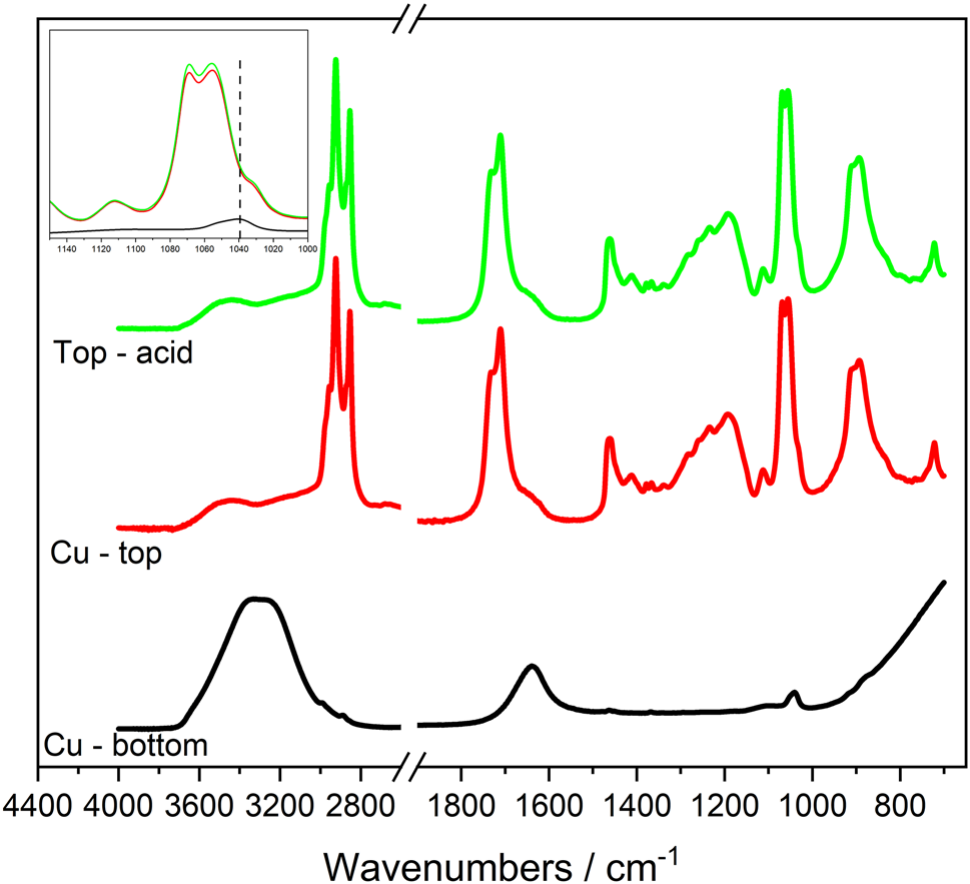
ATR-FTIR spectra of samples prepared with 1 M lauric acid, and 3:7 THF: 100 mM CuSO_4_ in water, at circum-neutral and acidic pH. These samples were separated into a top and a bottom phase. The color coding is as follows: bottom layer (black), top layer (red) and top layer at acidic pH (green). Red and green lines are identical in shape and intensity. Inset shows the sulfate absorbing region, indicating that there is no sulfate in the top layer of separated THF-water mixtures, in the presence of copper.

The data show that laurate ions (present at circum-neutral pH) act as carriers for Cu^2+^, transporting it into the THF phase. Cu^2+^remain in the THF-rich phase over a period of approximately three weeks, after which they return into the water phase. These results indicate that Cu^2+^migration into the THF-rich phase is a transient phenomenon, which would however allow removal of copper from water. We conducted ATR-FTIR experiments to probe copper-lauric acid interactions. Figure 7 shows the ATR-FTIR spectra of the top and bottom phase of samples prepared with 1 M lauric acid and either 3:7 THF:100 mM CuSO_4_ mixtures (using water at circum-neutral pH) or 3:7 THF:water mixtures (using water at acidic pH). The reason for using water at acidic pH in the absence of copper salts is to have a largely clear top, THF-rich layer. The top layer is also largely clear at circum-neutral pH, in the presence of copper salts (Fig. 6). When samples are prepared with CuSO_4_, the data indicate its presence in the bottom layer, whereas the spectrum of the top layer is identical with and without copper (inset shows sulfate region in Fig. 7). Also, the signatures of Cu-laurate complexes and sulfates are not detected in ATR-FTIR spectra. Nonetheless, the top layer has a blue discoloration, as highlighted above (Fig. 6). Therefore, the data suggest that laurate ions release free copper ions in the top THF-rich phase, rather than forming stable complexes with it. Previous studies report that metal ion transport into organic phases can be mediated by carboxylate ions^112–114^, in agreement with our hypothesis and results. After dissociating from laurate ions, Cu^2+^ions eventually return to the water phase, as indicated earlier.

Finally, before discussing Cu^2+^ partitioning into water-immiscible solvents such as toluene and hexane, note that Cu^2+^ also partitions into dioxane at circum-neutral pH (although lauric acid-induced dioxane-water separation is poor, Figs. S8–S11).

Copper partitioning between toluene and water was examined through bottle tests conducted using 30 mM CuCl_2_ or CuSO_4_ solutions, toluene, and either lauric acid alone or a solution of lauric acid in canola oil (added as a 23 mM lauric acid in canola oil solution), when using water with initial pH = 13 (adjusted with 10 mM NaOH). Dispensing lauric acid in canola oil solutions would be useful for groundwater remediation, to deliver lauric acid to the polluted zones. When treating water at the surface, neat lauric acid can be used instead. Bottle tests show that Cu^2+^ initially partitions in the toluene phase in the presence of lauric acid, when using water with initial pH = 13 (adjusted with NaOH, Figs. S12, S13, supporting information file). After 24 h, Cu^2+^ ions segregate in a layer at the toluene-water interface (Fig. S14, supporting information file). Similar results are also obtained with hexane (supporting information, Fig. S15). This does not occur in the absence of either lauric acid or without NaOH (Fig. S13, supporting information). As discussed earlier, the pKa of lauric acid is ≈5^90^. At basic pH, lauric acid would be dissociated and it would therefore be able to interact with copper cations (e.g., through electrostatic interactions).

ICP tests conducted on the water phase was used to quantify the percent removal of Cu^2+^ ions from CuCl_2_ and CuSO_4_ solutions (Tables 7, 8). Removal was greatest (≈60%) with initial concentrations of 10 mM copper ions in water, when using 1:1 0.25 M lauric acid in canola oil:toluene as the oil phase. Percent removal efficiency decreases with 10 mM copper ions in water, when the lauric acid concentration is kept constant. This is likely because less copper can bind to lauric acid, when the ratio of lauric acid to copper ions decreases. Future research will focus on identifying compounds which enable greater copper ion removal from water.

**Table 7:** ICP analyses of the water phase of samples prepared starting with 30 mM CuCl_2_ and 10 mM CuCl_2_, using toluene and lauric acid solutions in canola oil.

Water phase (5 mL)	Oil phase (total volume in brackets)	Cu^2+^ concentration in water after treatment (mM) [Average % Cu^2+^ removal]
30 mM CuCl_2_ + 10 mM NaOH	1:1 toluene: 0.25 M lauric acid in canola oil solution (2 mL)	17.2 ± 0.2 [42.8]
10 mM CuCl_2_ + 10 mM NaOH	1:1 toluene: 0.25 M lauric acid in canola oil solution (2 mL)	3.9 ± 0.1 [61.1]
30 mM CuCl_2_ + 10 mM NaOH	1:4 toluene: 0.25 M lauric acid in canola oil solution (5 mL)	13.3 ± 1.1 [55.7]

The volumes used and the lauric acid concentrations are as indicated in the table. Partitioning of copper ions in the toluene phase with lauric acid and a base is also achieved without using canola oil, as qualitatively assessed based on the discoloration of the toluene phase (Fig. S13, supporting information). Measurements were done after 24 h equilibration.

**Table 8:** ICP analyses of the water phase of samples prepared starting with 30 mM CuSO_4_ and 10 mM CuSO_4_, using toluene and lauric acid solutions in canola oil.

Water phase (5 mL)	Oil phase (total volume in brackets)	Cu^2+^ concentration in water after treatment (mM) [Average % Cu^2+^ removal]
30 mM CuSO_4_ + 10 mM NaOH	1:1 toluene: 0.25 M lauric acid in canola oil solution (2 mL)	17.6 ± 2.5 [41.4]
10 mM CuSO_4_ + 10 mM NaOH	1:1 toluene: 0.25 M lauric acid in canola oil solution (2 mL)	4.2 ± 0.2 [57.9]
30 mM CuSO_4_ + 10 mM NaOH	1:4 toluene: 0.25 M lauric acid in canola oil solution (5 mL)	15.4 ± 2.0 [48.8]

The volumes used and the lauric acid concentrations are as indicated in the table. Partitioning of copper ions in the toluene phase with lauric acid and a base is also achieved without using canola oil, as qualitatively assessed based on the discoloration of the toluene phase (Fig. S13, supporting information). Measurements were done after 24 h equilibration.

**Figure 8:**
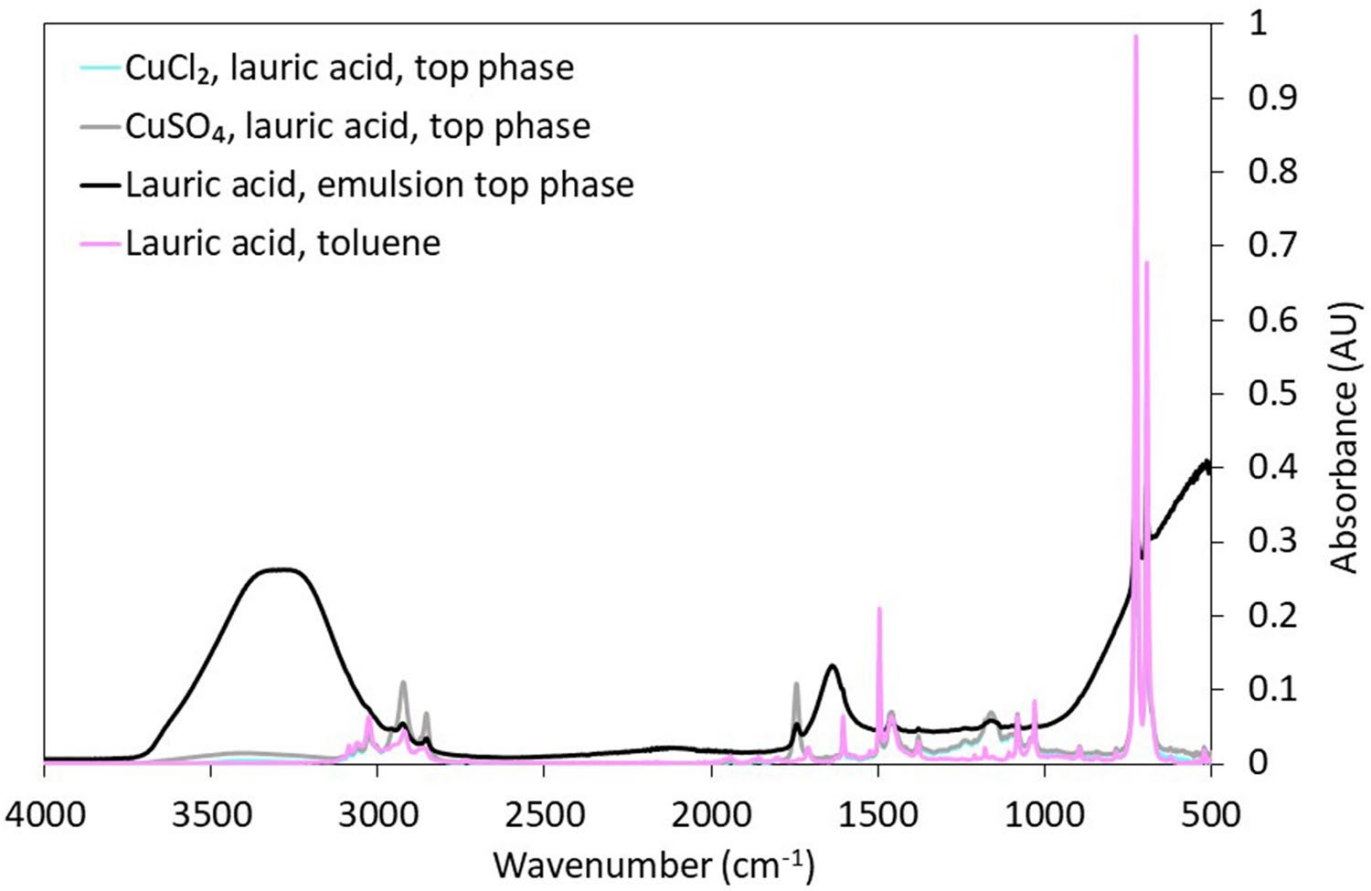
ATR-FTIR spectra of samples prepared with lauric acid in canola oil and toluene and DI water with 10 mM NaOH, with or without copper salts, and with lauric acid in toluene alone. ATR-FTIR spectra were collected for the top phase (i.e., the oil phase) of samples prepared using 5 mL of either 30 mM CuCl_2_ or CuSO_4_ solutions, and 2 mL of oil (containing 1:1 toluene to 0.25 M lauric in canola oil solution).

As discussed earlier, Cu^2+^ and laurate ions likely interact through physical interactions (e.g., electrostatic interactions), causing Cu^2+^ to partition at the toluene-water interface. The ATR-FTIR spectra of the oil phase of samples containing either 30 mM CuCl_2_ or CuSO_4_, and 10 mM NaOH (relative to the water phase), lauric acid in canola oil and toluene are similar to those of samples containing lauric acid in toluene, with the exception of the peak at ≈1160 cm^−1^ (Fig. 8). This peak is also observed in the top phase of samples prepared with water and 10 mM NaOH, 0.25 M lauric acid in canola oil and toluene, without Cu^2+^. Therefore, it is not representative of chemical bonding between Cu^2+^ and lauric acid or laurate ions. New peaks do not appear in the spectra of samples containing both lauric acid and copper salts. Therefore, ATR-FTIR data do not highlight any chemical bonding between Cu^2+^ and lauric acid, in agreement with the results obtained with THF.

Note the marked ATR-FTIR -OH scissor and -OH stretch absorbance peaks at ≈1600 cm^−1^ and ≈3400 cm^−1^ in the ATR FTIR spectra of samples prepared with water and 10 mM NaOH, 0.25 M lauric acid in canola oil and toluene (without copper salts). These peaks are absent in the oil phase of samples which also contain copper salts, indicating negligible water content. This result indicates that water in toluene emulsions are more stable with than without copper salts. Emulsion stability is further discussed in the following section (“Emulsion stability with copper salts”).

### Emulsion stability with copper salts

The goal of this study is to use lauric acid to separate water from toluene and copper, and THF and copper. Stable emulsions of either THF or toluene in water would entrain organics solvents and copper (partitioned inside them) into the water phase. Unless filters are used, this would hinder water purification. “Flow test experiments (injectable filters)” will discuss injectable filters, which can be used in conjunction with lauric acid for the treatment of copper and toluene pollution in groundwater. Stable water in oil emulsions would be less problematic, although they are not desirable because they would lead to larger volumes of liquid waste following treatment.

Water in THF and THF in water emulsions are not stable with THF and either CuCl_2_ or CuSO_4_, as shown for instance in Fig. 6 (where mixtures are separated into free phases). As a result, there should be limited entrainment of Cu^2+^ in the water phase.

**Figure 9:**
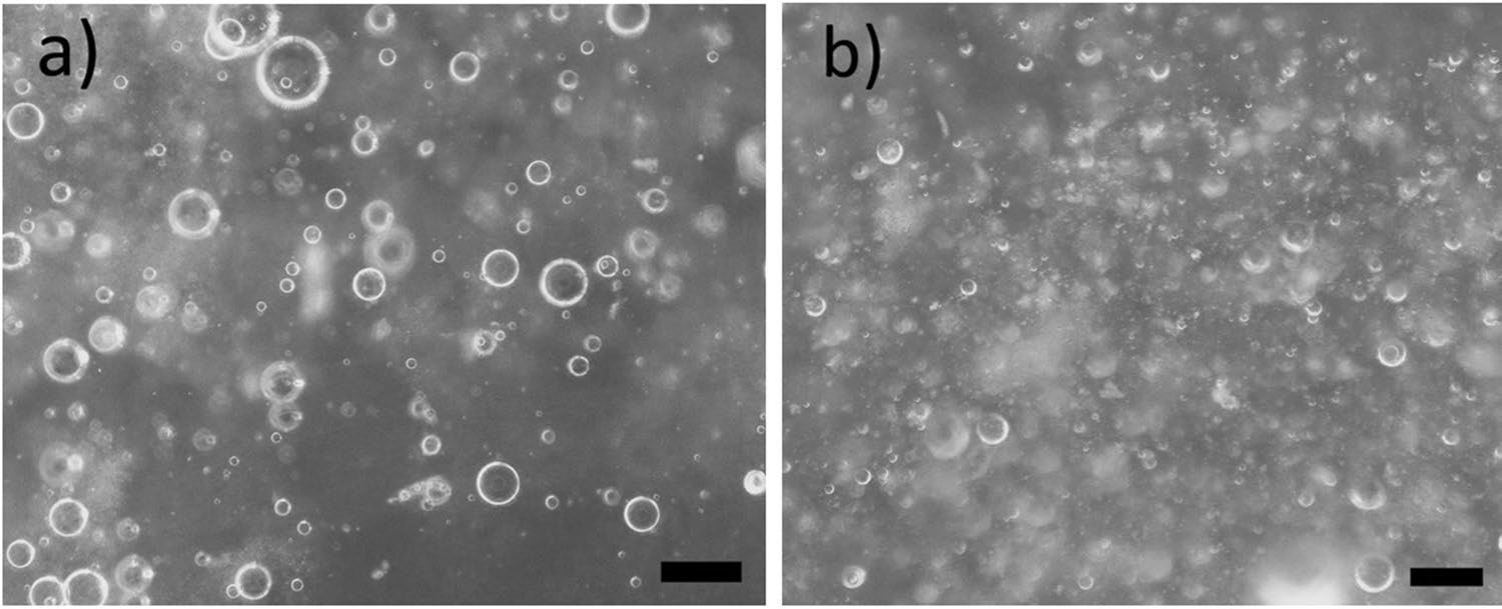
Optical microscopy images of water in oil emulsions sampled following bottle tests conducted using toluene (10% v/v), 0.25 M lauric acid in canola oil (10% v/v) and either 30 mM CuCl_2_ solutions (70% v/v, a) or 30 mM CuSO_4_ solutions (70% v/v, b), with 10 mM NaOH. The scalebar is 100 μm.

With 50% water and 50% toluene (without canola oil), some toluene remains emulsified in water after a day, as indicated by residual turbidity of the water phase (Fig. S13, supporting information file). Similar results are obtained with canola oil (Fig. S12, supporting information file). Recall that canola oil is used to enable the delivery of lauric acid into polluted aquifers. Over short time intervals, water in oil and oil in water emulsions are observed for samples prepared with either 30 mM CuCl_2_ or CuSO_4_ aqueous solutions, toluene and lauric acid in canola oil solutions (cf. optical microscopy images, Fig. 9). Toluene droplets in water can be excluded using filters, as we will further discuss in the following section (“Flow test experiments (injectable filters)”). Water in oil emulsions are promptly destabilized with CuSO_4_, whereas sparse oil in water droplets are observed with CuCl_2_ (image not shown). After 24 h, water in oil emulsions are also not observed with CuCl_2_. After this time period, flocs segregate at the oil–water interface (e.g., Fig. S14, supporting information).

The interfacial tension measured with lauric acid at the water-canola oil + toluene interface is 10.30 ± 0.18 mN/m, with 10 mM NaOH. The interfacial tension of lauric acid decreases to 27.32 ± 0.03 mN/m with CuSO_4_ and NaOH, confirming that Cu^2+^ interacts with lauric acid (likely through electrostatic interactions), forming flocs which are less effective at stabilizing emulsions than free laurate ions.

**Figure 10:**
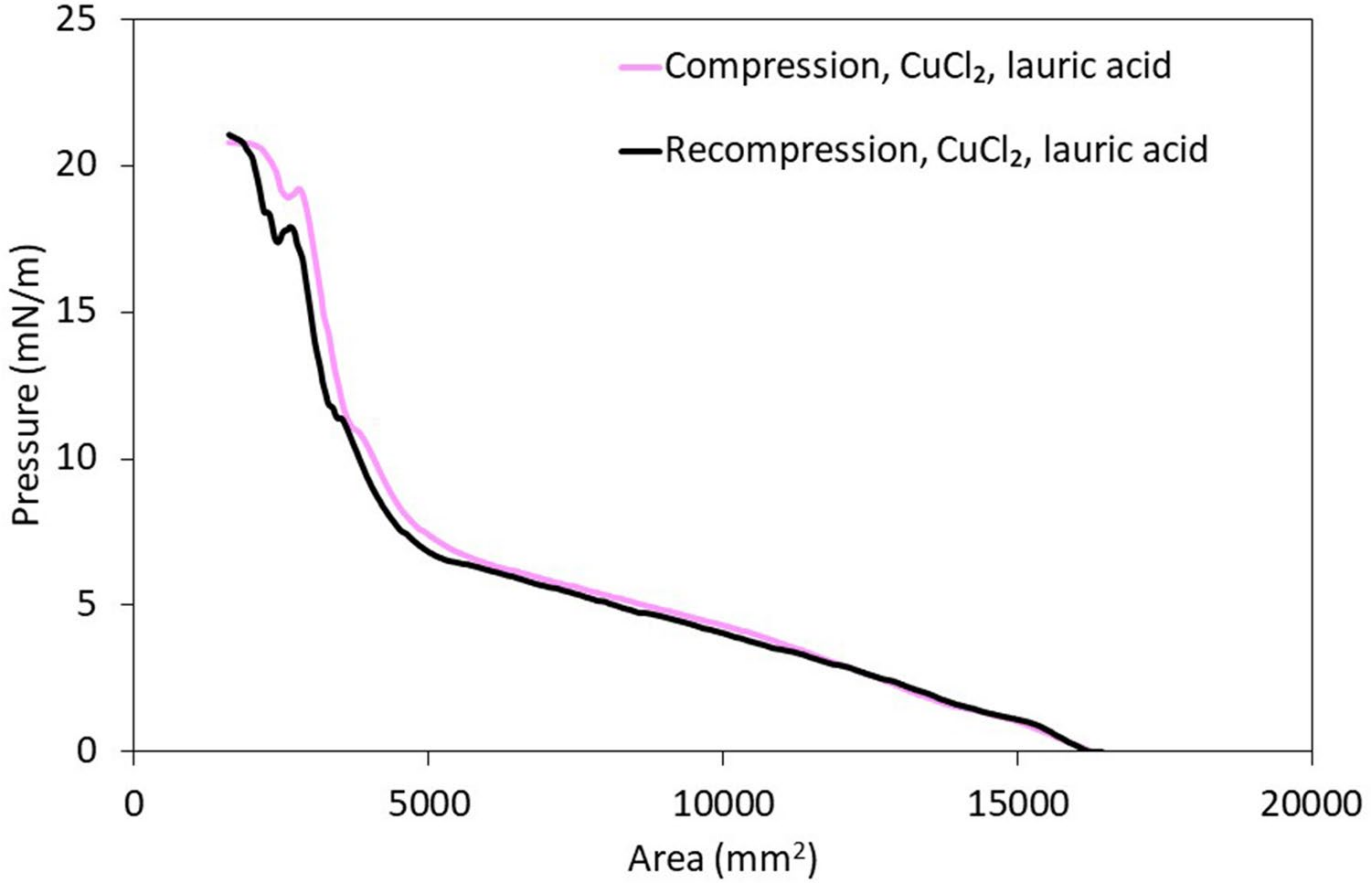
Sample compression isotherm measured at the oil water interface. The aqueous and oil phases were obtained starting from samples prepared with 2:1 toluene: 0.25 M lauric in canola oil solution and copper salt solutions (30 mM CuCl_2_ + 10 mM NaOH or 30 mM CuSO_4_ + 10 mM NaOH), as described in “Static interfacial tension measurement”. Samples were separated and each phase was re-introduced in the trough, to create a planar interface.

Compressional rigidity is also correlated to emulsion stability^18^. Compression isotherms measured with lauric acid and CuSO_4_ are largely flat (data not shown), indicating that either films are soft or that interfacially active species rapidly desorb upon compression^109^. Either scenario would lead to poor emulsion stability^109^. Note that other factors also affect emulsion stability^109^. As an example, flocs can contribute steric repulsion between droplets and stabilize them through Pickering stabilization mechanisms^23^. Pickering stabilization mechanisms can account for the stability of emulsions observed with lauric acid and CuSO_4_ over short time periods. Compression isotherms measured with lauric acid and CuCl_2_ indicate that interfacial films are rigid, as demonstrated by the increase in pressure (19 ± 3 mN/m, Fig. 10). When films were re-compressed immediately after the first compression, the re-compression isotherm is similar to the first compression. This indicates that interfacially active species either do not desorb upon compression, or they rapidly re-adsorb^18^. Either scenario would promote emulsion stability, explaining why emulsions were more stable with CuCl_2_ than with CuSO_4_^18^. Nonetheless, emulsions were not observed after 24 h even with CuCl_2_, as highlighted above. Rapid emulsion separation can be achieved even in aquifers using injectable filters, as discussed in “Flow test experiments (injectable filters)”.

### Flow test experiments (injectable filters)

**Figure 11:**
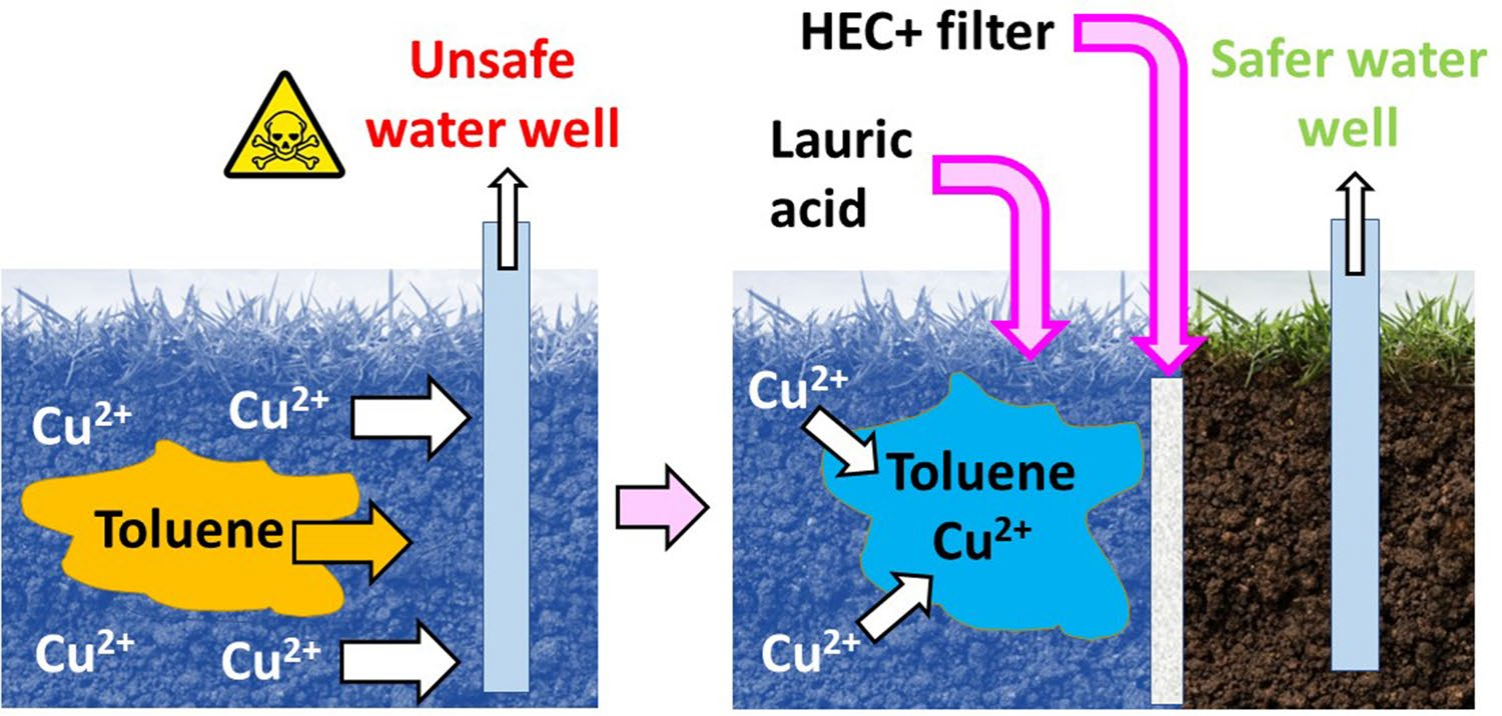
Schematics of the proposed approach, which combines HEC + injectable filters and lauric acid to simultaneously prevent copper ion and toluene migration. Once the flow of toluene and copper is arrested upstream of the filters, these contaminants can be extracted from the aquifer (e.g., using a pumping well). Note that while HEC + filters retain toluene in which Cu^2+^ partitions, they allow water flow. Also note that while mixing lauric acid in canola oil is required to enable its injection in polluted aquifers, lauric acid can be introduced in wastewater to be treated at the surface in its dry form (e.g., as a powder).

Flow tests experiments are used to investigate the effectiveness of injectable filters in removing copper from water co-polluted by copper and toluene, when used in conjunction with lauric acid. These filters would enable the simultaneous separation of copper and toluene from water, preventing them from migrating downstream. Pollutants could then be extracted upstream from the filter (Fig. 11).

Our previous studies showed that injectable filters could retain hydrophobic solvents such as toluene and hexane^51^, and that HEC + injectable filters retained diesel droplets emulsified by bacterial biosurfactants^81^. Here, HEC + injectable filters retain toluene containing Cu^2+^ ions, as shown by ICP-OES analyses (Table 9). Copper ion removal achieved using filters is comparable to bottle test experiments (≈60%, starting from 30 mM Cu^2+^), even without equilibrating for 24 h. This result indicates that filters aid in the simultaneous removal of copper ions and toluene.

**Table 9:** ICP analyses of the water phase of samples prepared starting with 30 mM CuSO_4_ or CuCl_2_, using toluene and lauric acid solutions in canola oil.

Water phase composition (5 mL)	Oil phase (2 mL)	Cu^2+^ concentration in water after treatment (mM) [Cu^2+^ removal from water]
30 mM CuCl_2_ + 10 mM NaOH	1:1 toluene: 0.25 M lauric in canola oil solution	12.2 ± 0.8 [59.5]
30 mM CuSO_4_ + 10 mM NaOH	1:1 toluene: 0.25 M lauric in canola oil solution	12.4 ± 1.7 [58.8]

Measurements were done immediately after filtering the samples through the injectable filters.

In contrast, these filters cannot exclude THF separated from water by lauric acid, because of its higher polarity compared to toluene. Therefore, lauric acid cannot be used for the in situ separation of water from THF and copper ions. In contrast, lauric acid would be better suited to treat water polluted by copper and THF at the surface, for instance after its extraction from polluted aquifers using pump and treat.

## Conclusions

Lauric acid can be used to purify water polluted with THF at ambient temperature (20 °C), with minimal energy costs. At the onset of THF-water separation and with > 90% THF (v/v, relative to water), lauric acid self-assembles into reverse micelles. These reverse micelles are ≈25 Å in size and host water in their interior, as shown by SAXS. They swell with increasing water content, ultimately leading to free phase separation. With 1 M lauric acid and 7:3 THF:water mixtures, the purity of the water phase is 87% at either acidic, semi-neutral or basic pH (as shown by ^1^H NMR). Separation efficiency decreases at lower lauric acid concentrations, and is 76% with 0.125 M lauric acid. Therefore, our facile treatment approach finds potential applications for the treatment of either wastewater or groundwater.

Lauric acid also removes copper from water co-polluted with either THF or toluene. It interacts with copper ions through physical interactions (e.g., electrostatic interactions) and causes their migration into the organic phase. Model sandy aquifers co-polluted with toluene and Cu^2+^ were treated with lauric acid, and HEC + semi-permeable barriers were placed downstream of the pollutant plume. These barriers retain both toluene and ≈50% Cu^2+^. Therefore, they have the potential to offset the risk of pollutant migration and aid in the protection of downstream receptors. Extraction of co-contaminants upstream of the filters can be achieved using pumping wells, enabling their simultaneous removal from aquifers.

Future research will focus on removing additional ions from water. We will also focus on improving the purity of the water phase separated from water miscible solvents and ions using other amphiphilic molecules.

## Supplementary Information

Supplementary Information.

## Data Availability

The datasets used and/or analysed during the current study available from the corresponding author on reasonable request.
