# Supplementary material for: Decontamination of water co-polluted by copper, toluene and tetrahydrofuran using lauric acid
Source: Sci Rep. 2022 Sep 22;12:15832. doi: 10.1038/s41598-022-20241-4 (PMC9500063; doi:10.1038/s41598-022-20241-4)
Supplement: Supplementary file 1 — Supplementary Information. [file 41598_2022_20241_MOESM1_ESM.docx]

**Supporting Information**

**Decontamination of water co-polluted by copper, toluene and tetrahydrofuran using lauric acid**

Laura Earnden^1^, Alejandro G. Marangoni^2^, Thamara Laredo^3^, Jarvis Stobbs^2,4^, Tatianna Marshall^1^, Erica Pensini^1^*

^1^ University of Guelph, School of Engineering, 50 Stone Road East, Guelph (ON), N1G 2W1, Canada

^2^University of Guelph, Food Science Department, 50 Stone Road East, Guelph (ON), N1G 2W1, Canada

^3^Lakehead University, Chemistry Department, 500 University Ave, Orillia (ON), L3V 0B9, Canada

^4^ Canadian Light Source Synchrotron, 44 Innovation Boulevard, Saskatoon (SK), S7N 2V3, Canada

*Corresponding author. Email: [epensini@uoguelph.ca](mailto:epensini@uoguelph.ca), phone: +1 519-824-4120 ext. 56746, address: University of Guelph, Room 2525 Richards Bld., 50 Stone Road East, Guelph, ON N1G 2W1

**Summary**

This supporting information file contains images of bottle tests conducted with lauric acid at different pH values, using 3:7 THF:water mixtures and toluene-water mixtures (Figs. SI.1-SI.2). This file also contains fits of the H-bonding peak of water in the presence and in the absence of lauric acid (Fig.SI.2) and the ATR-FTIR spectra of THF and THF-lauric acid solutions (Fig.SI.3-SI.5). Fig. SI.6 and SI.7 refer to experiments conducted to prove that lauric acid also separate dioxane and isopropyl alchol from water. Figs. SI.8-SI.15 show optical microscopy images and bottle tests conducted using lauric acid, copper salts, toluene and hexane. The Gaussian model fits to XRD patters are also provided (Table SI.4).

1. **Bottle tests conducted with lauric acid, THF and water**


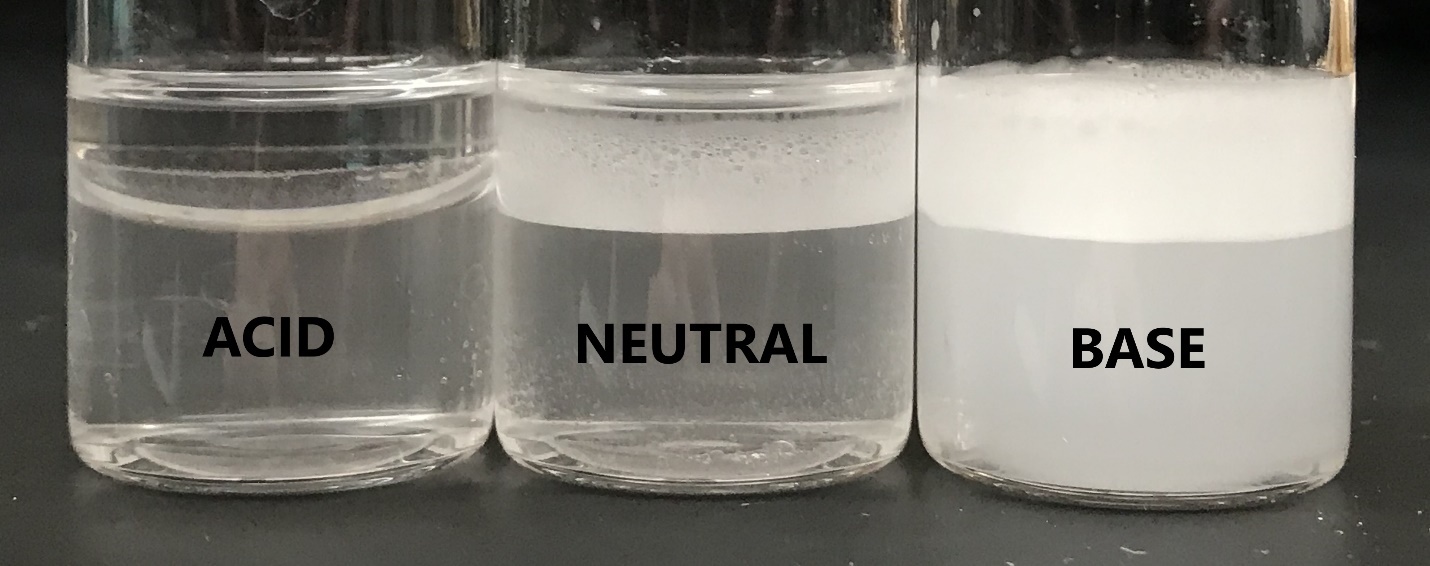


**Figure SI.1** Emulsions stabilized by 0.125 M lauric acid in toluene-water mixtures (containing 30% toluene and 70% water, v/v), at either acidic (left), circum-neutral (center) or basic (left) pH. The image is captured 30 min after mixing. Note the marked turbidity of both the top layer (toluene) and bottom layer (water) at basic pH, indicating water in toluene and toluene in water stable emulsions.


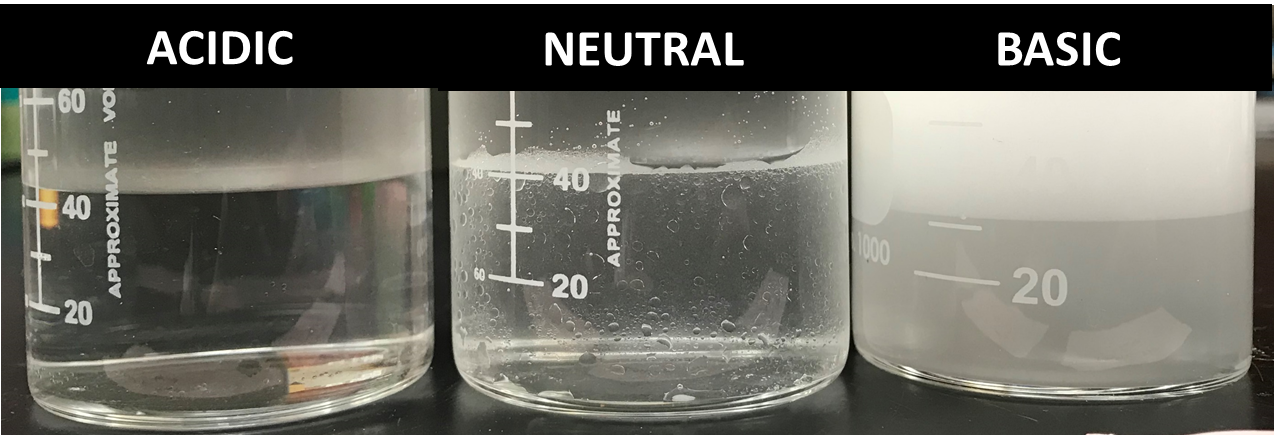


**Figure SI.2** Separation between THF and water at acidic (left), circum-neutral (center) and acidic (left) pH, 5 min after mixing, with 1 M lauric acid. The THF-water mixture contains 30% THF v/v.

1. **Analysis of the H-bond region of ATR-FTIR spectra of water with lauric acid**

**Table SI.1** Parameters for the sample fit shown in Fig. SI.3.

|  | Water neutral pH | Water acidic pH |
| --- | --- | --- |
| Sum of two Gaussians |  |  |
| Best-fit values |  |  |
| Amplitude1 | 0.3649 | 0.4030 |
| Mean1 | 3184 | 3186 |
| SD1 | 93.83 | 96.83 |
| Amplitude2 | 0.9011 | 0.8827 |
| Mean2 | 3353 | 3359 |
| SD2 | 148.6 | 146.1 |
| Std. Error |  |  |
| Amplitude1 | 0.01736 | 0.01862 |
| Mean1 | 1.008 | 1.262 |
| SD1 | 1.474 | 1.404 |
| Amplitude2 | 0.008625 | 0.009694 |
| Mean2 | 2.482 | 2.691 |
| SD2 | 1.041 | 1.104 |
| 95% CI (asymptotic) |  |  |
| Amplitude1 | 0.3309 to 0.3989 | 0.3665 to 0.4395 |
| Mean1 | 3182 to 3186 | 3183 to 3188 |
| SD1 | 90.94 to 96.72 | 94.07 to 99.58 |
| Amplitude2 | 0.8842 to 0.9180 | 0.8637 to 0.9017 |
| Mean2 | 3348 to 3358 | 3353 to 3364 |
| SD2 | 146.6 to 150.7 | 144.0 to 148.3 |
| Goodness of Fit |  |  |
| Degrees of Freedom | 2339 | 2339 |
| R squared | 0.9990 | 0.9989 |
| Sum of Squares | 0.3710 | 0.3888 |
| Sy.x | 0.01259 | 0.01289 |
| Constraints |  |  |
| Amplitude1 | Amplitude1 > 0 | Amplitude1 > 0 |
| Mean1 | Mean1 > 0 | Mean1 > 0 |
| SD1 | SD1 > 0 | SD1 > 0 |
| Amplitude2 | Amplitude2 > 0 | Amplitude2 > 0 |
| Mean2 | Mean2 > 0 | Mean2 > 0 |
| SD2 | SD2 > 0 | SD2 > 0 |
|  |  |  |
| Number of points |  |  |
| # of X values | 3112 | 3112 |
| # Y values analyzed | 2345 | 2345 |

**Table SI.2** Parameters for the sample fit shown in Fig. SI.3. The analysis refers to the bottom-rich phase separated by lauric acid.

|  | 30% THF, 70% water, lauric acid, neutral pH | 30% THF, 70% water, lauric acid, acidic pH |
| --- | --- | --- |
| Sum of two Gaussians |  |  |
| Best-fit values |  |  |
| Amplitude1 | 0.4270 | 0.4366 |
| Mean1 | 3189 | 3191 |
| SD1 | 98.70 | 99.09 |
| Amplitude2 | 0.8777 | 0.8731 |
| Mean2 | 3367 | 3369 |
| SD2 | 140.8 | 140.2 |
| Std. Error |  |  |
| Amplitude1 | 0.005716 | 0.006556 |
| Mean1 | 0.4999 | 0.5796 |
| SD1 | 0.4068 | 0.4535 |
| Amplitude2 | 0.003134 | 0.003627 |
| Mean2 | 0.8209 | 0.9448 |
| SD2 | 0.3337 | 0.3827 |
| 95% CI (asymptotic) |  |  |
| Amplitude1 | 0.4158 to 0.4382 | 0.4238 to 0.4495 |
| Mean1 | 3188 to 3190 | 3190 to 3192 |
| SD1 | 97.90 to 99.50 | 98.20 to 99.98 |
| Amplitude2 | 0.8716 to 0.8839 | 0.8660 to 0.8802 |
| Mean2 | 3365 to 3368 | 3367 to 3370 |
| SD2 | 140.1 to 141.4 | 139.5 to 141.0 |
| Goodness of Fit |  |  |
| Degrees of Freedom | 23444 | 16395 |
| R squared | 0.9989 | 0.9990 |
| Sum of Squares | 3.860 | 2.457 |
| Sy.x | 0.01283 | 0.01224 |
| Constraints |  |  |
| Amplitude1 | Amplitude1 > 0 | Amplitude1 > 0 |
| Mean1 | Mean1 > 0 | Mean1 > 0 |
| SD1 | SD1 > 0 | SD1 > 0 |
| Amplitude2 | Amplitude2 > 0 | Amplitude2 > 0 |
| Mean2 | Mean2 > 0 | Mean2 > 0 |
| SD2 | SD2 > 0 | SD2 > 0 |
|  |  |  |
| Number of points |  |  |
| # of X values | 31120 | 31120 |
| # Y values analyzed | 23450 | 16401 |

**Table SI.3** Parameters for the sample fit shown in Fig. SI.3.

|  | 30% THF, 70% water, neutral pH | 30% THF, 70% water, acidic pH |
| --- | --- | --- |
| Sum of two Gaussians |  |  |
| Best-fit values |  |  |
| Amplitude1 | 0.3649 | 0.4030 |
| Mean1 | 3184 | 3186 |
| SD1 | 93.83 | 96.83 |
| Amplitude2 | 0.9011 | 0.8827 |
| Mean2 | 3353 | 3359 |
| SD2 | 148.6 | 146.1 |
| Std. Error |  |  |
| Amplitude1 | 0.01736 | 0.01862 |
| Mean1 | 1.008 | 1.262 |
| SD1 | 1.474 | 1.404 |
| Amplitude2 | 0.008625 | 0.009694 |
| Mean2 | 2.482 | 2.691 |
| SD2 | 1.041 | 1.104 |
| 95% CI (asymptotic) |  |  |
| Amplitude1 | 0.3309 to 0.3989 | 0.3665 to 0.4395 |
| Mean1 | 3182 to 3186 | 3183 to 3188 |
| SD1 | 90.94 to 96.72 | 94.07 to 99.58 |
| Amplitude2 | 0.8842 to 0.9180 | 0.8637 to 0.9017 |
| Mean2 | 3348 to 3358 | 3353 to 3364 |
| SD2 | 146.6 to 150.7 | 144.0 to 148.3 |
| Goodness of Fit |  |  |
| Degrees of Freedom | 2339 | 2339 |
| R squared | 0.9990 | 0.9989 |
| Sum of Squares | 0.3710 | 0.3888 |
| Sy.x | 0.01259 | 0.01289 |
| Constraints |  |  |
| Amplitude1 | Amplitude1 > 0 | Amplitude1 > 0 |
| Mean1 | Mean1 > 0 | Mean1 > 0 |
| SD1 | SD1 > 0 | SD1 > 0 |
| Amplitude2 | Amplitude2 > 0 | Amplitude2 > 0 |
| Mean2 | Mean2 > 0 | Mean2 > 0 |
| SD2 | SD2 > 0 | SD2 > 0 |
|  |  |  |
| Number of points |  |  |
| # of X values | 3112 | 3112 |
| # Y values analyzed | 2345 | 2345 |


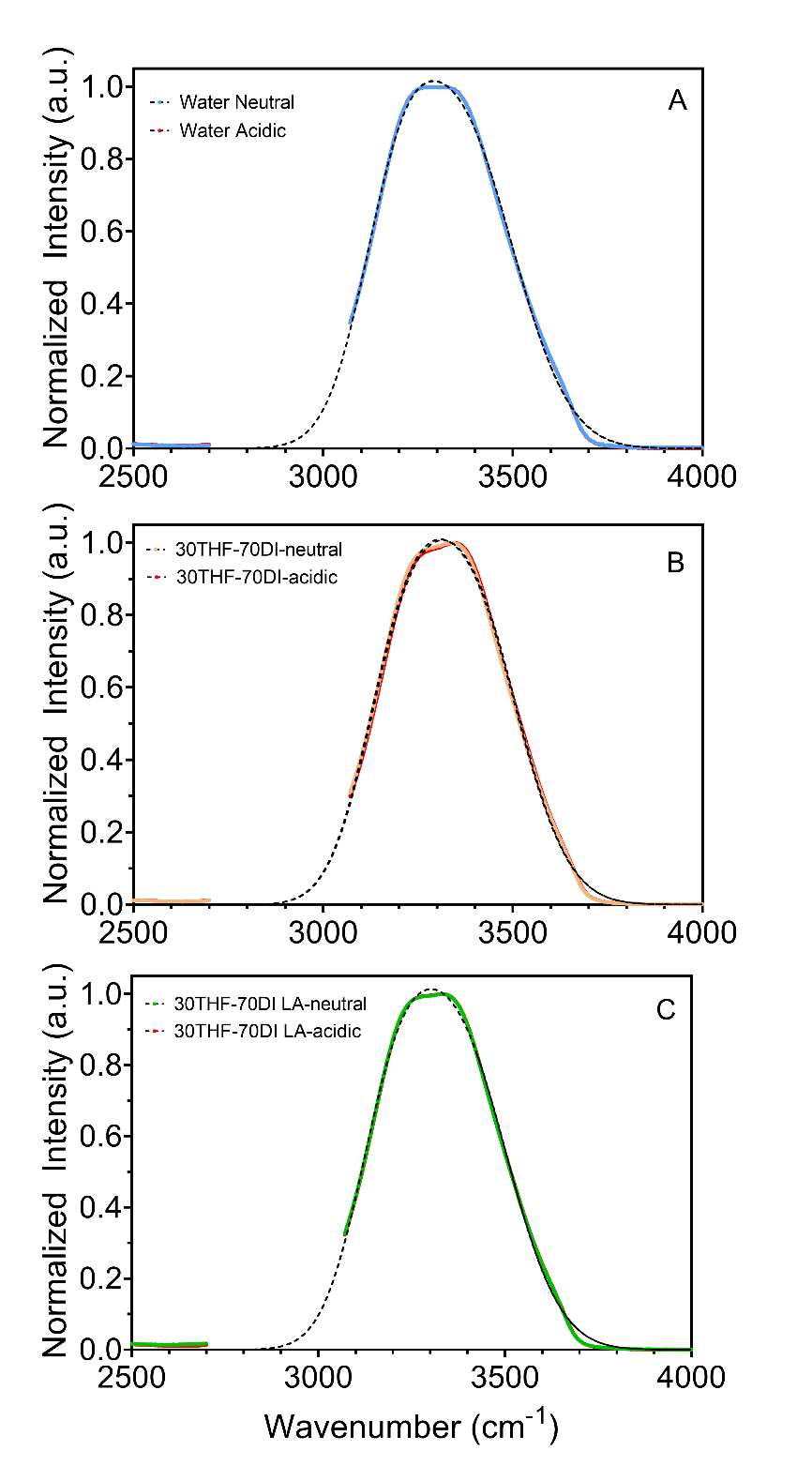


**Figure SI.3** Sample fit of the two Gaussian contributions to the ATR-FTIR peak for DI water, for 3:7 THF:DI water mixtures and for the bottom water-rich phase separated from 3:7 THF:DI water mixtures using 1 M lauric acid. The parameters for this fit are in Tables SI.1-SI.3.

1. **ATR-FTIR spectra of THF, lauric acid and lauric acid in THF**

The absorbance peak at ≈1732 cm^-1^ in the ATR-FTIR spectrum of lauric acid in pure THF corresponds to the stretch of free carbonyl groups of the carboxylic acid (Fig.SI.3, [1]), while the absorbance peak at ≈1710 cm^-1^ in the ATR-FTIR spectrum of lauric acid in THF corresponds to the stretch of H-bonded carbonyl groups of the carboxylic acid (Fig. SI.3, [2]). This peak is absent in the ATR-FTIR spectra of pure THF or lauric acid powder. In addition to H-bonding lauric acid, THF could also interact with the hydrophobic tails of lauric acid, facilitating dissolution.

**Figure SI.4** ATR-FTIR spectrum of lauric acid, 1.5 M lauric acid in THF, and THF alone.

1. **ATR-FTIR spectra of lauric acid in THF-water mixtures**


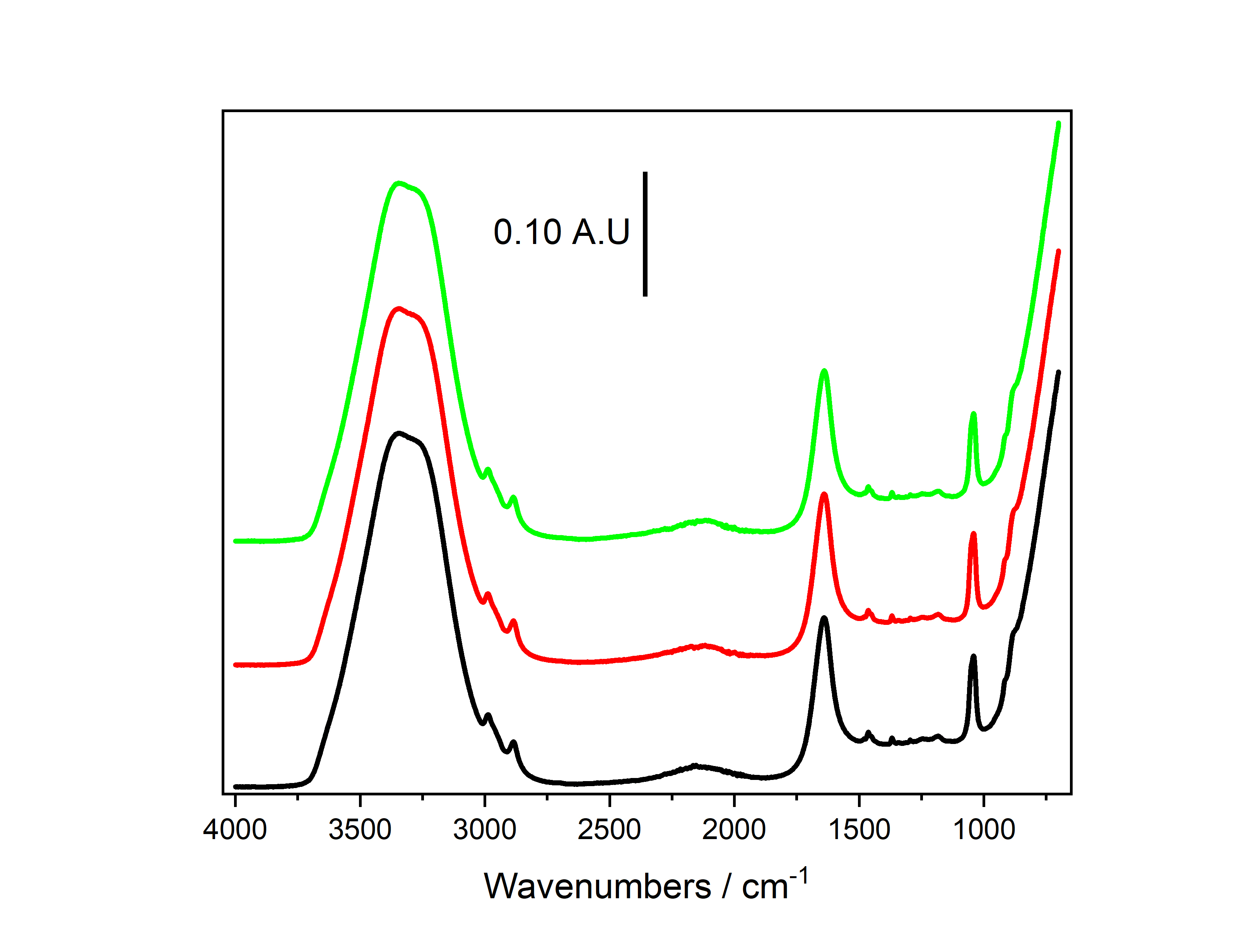


**Figure SI.5** ATR-FTIR spectra of samples prepared with 1 M lauric acid in 30:70 THF:water (volume ratio) using water at different pH values: acidic (black), circum-neutral (red) and basic (green). Spectra are identical in shape and intensity.


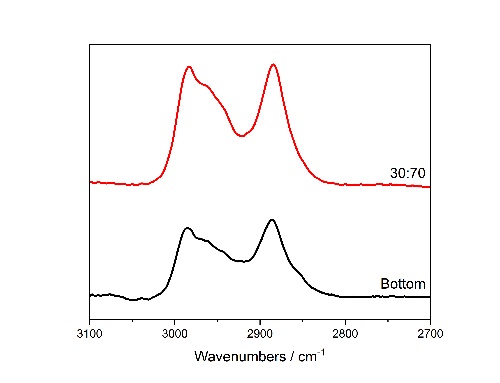

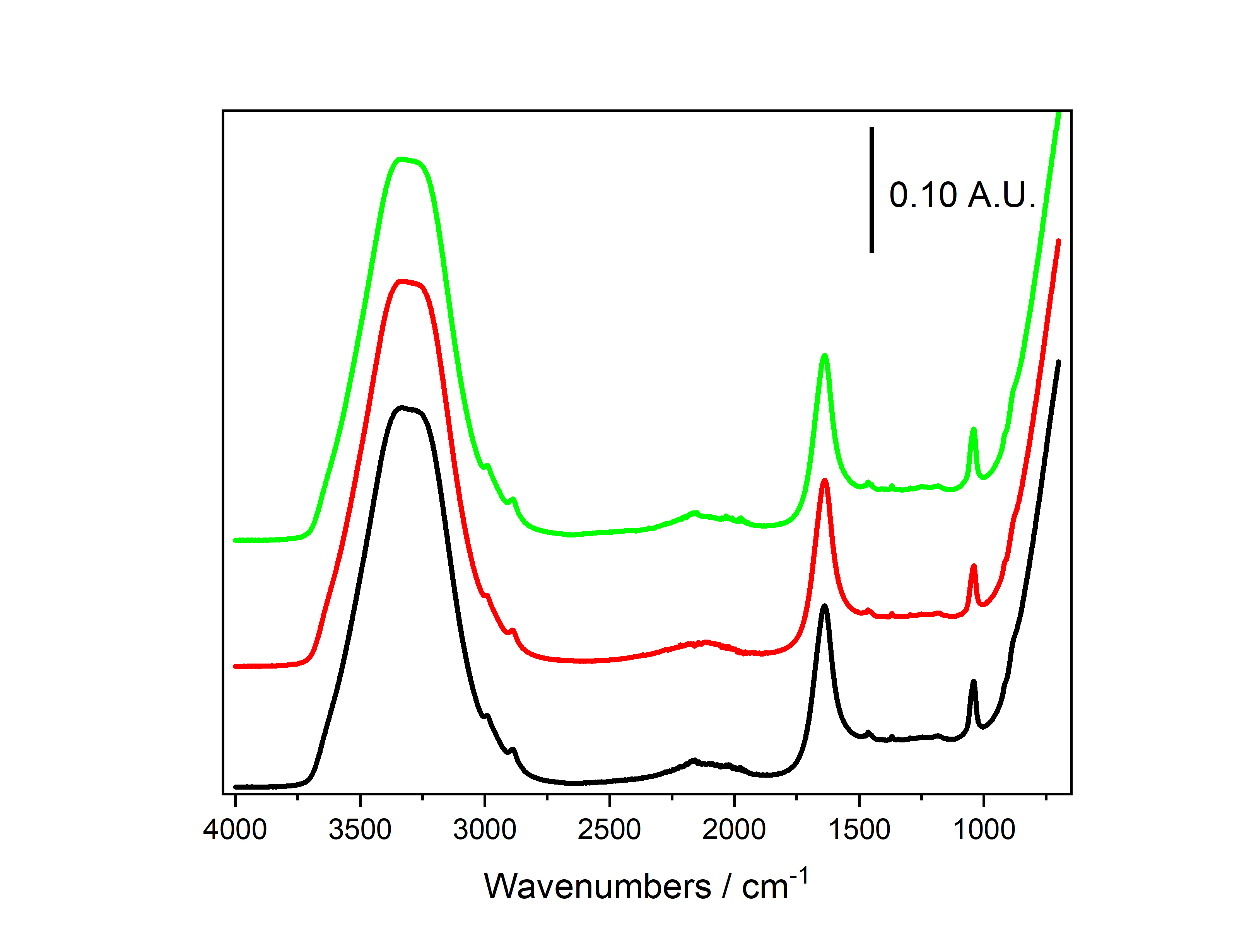


**Figure SI.6** ATR-FTIR spectra of the bottom phase (water-rich) separated using 1 M lauric acid, starting from 3:7 THF:water mixtures prepared with water at different pH values: acidic (black), circum-neutral (red) and basic (green). Spectra are identical in shape and intensity. With the exception of the region at 2900 cm^-1^ (inset), these spectra are also identical to those of 3:7 THF:water mixtures without lauric acid. This result indicated that lauric acid is not present in the bottom phase. Difference in intensities in the 2900 cm^-1^ region allowed for the estimation of THF in the bottom phase. Inset shows baseline corrected spectra of the 2900 cm^-1^ region for the bottom (black) phase of mixtures separated with lauric acid and for THF-water mixtures without lauric acid (red). From the value of the area under each curve, it was determined that the bottom layer still contains approximately 19% of THF, in line with NMR results.

1. **Separation between water, dioxane and isopropyl alchohol (IPA) by lauric acid**

**Figure SI.7** Separation between IPA and water with different lauric acid concentrations, and different IPA-water ratios. The percent of IPA provided in the legend refers to the percent of IPA relative to water (v/v). The deviation shown on the ordinate axis is calculated as (V_water,measured_-V_water,used_/V_water,used_)*100(%).


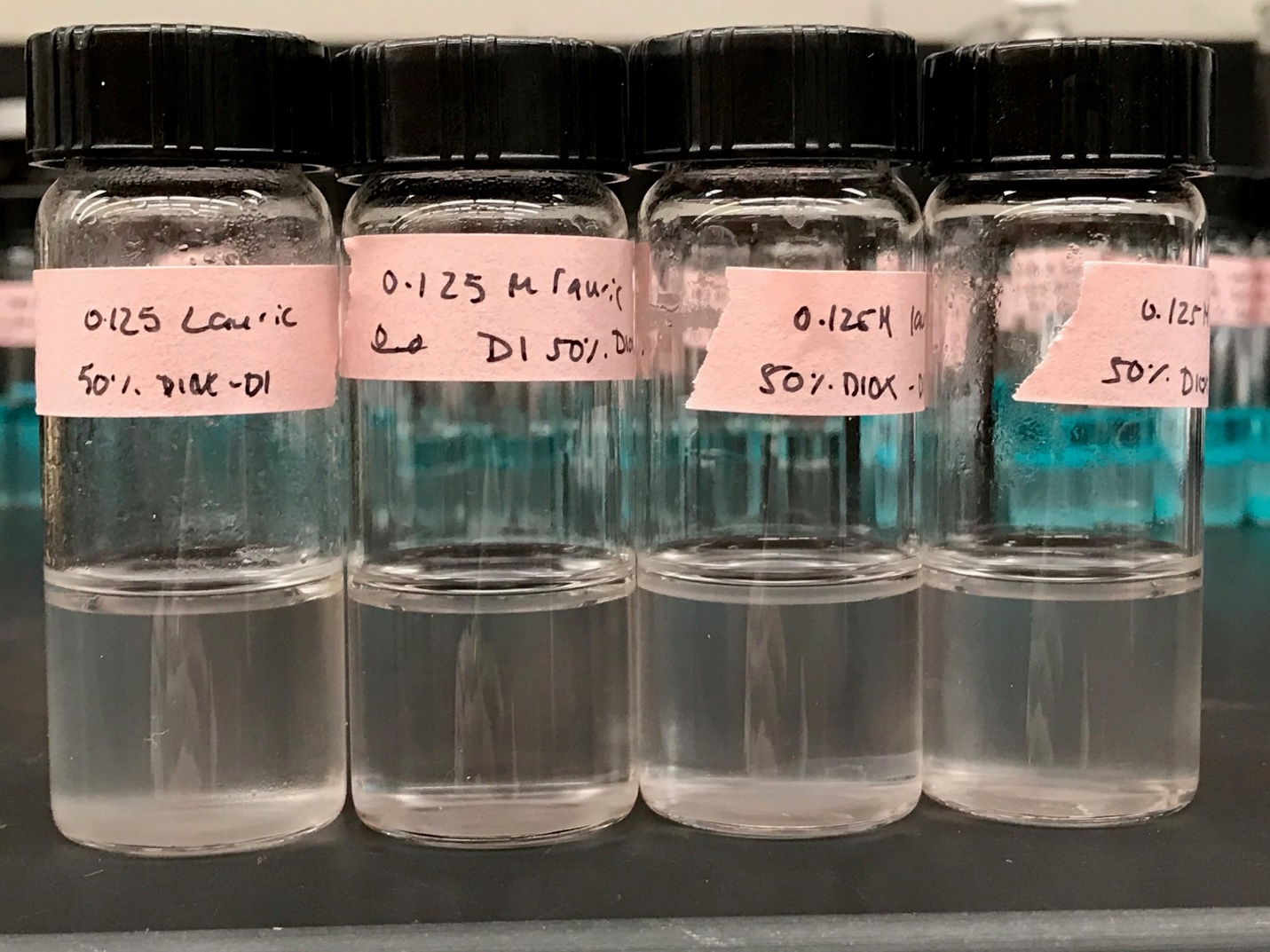


**Figure SI.8** Separation between dioxane and water with 0.125 M lauric acid, starting from a 50:50 water:dioxane ratio (v/v), with water at circum-neutral pH.

1. **Separation between water, THF and copper by lauric acid**


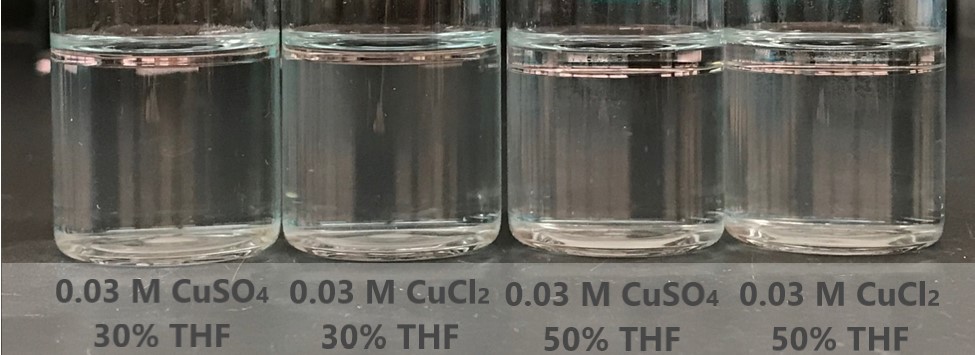


**Figure SI.9** Bottle tests conducted using 0.3 M CuCl_2_ and CuSO_4_, with 30% THF and 70% water, at pH=3. Note that the THF-rich layer does not have a blue discoloration, dissimilar to circum-neutral pH. This indicates that Cu^2+^ ions do not partition in THF at low pH.


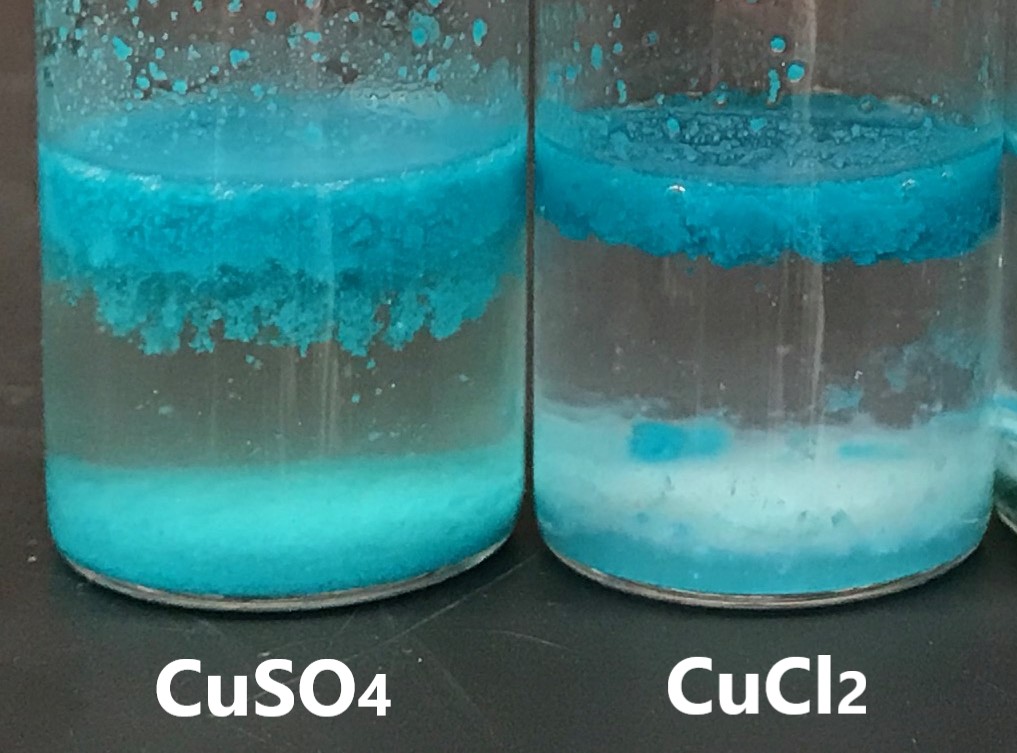


**Figure SI.10** Bottle tests conducted using 0.03 M CuCl_2_ (right) and CuSO_4_ (left), with 30% THF and 70% water (v/v), at pH=13. While part of the copper ions partitioned in THF, part of it precipitated out of solution. The white color of the precipitates suggests that lauric acid co-precipitates with copper.


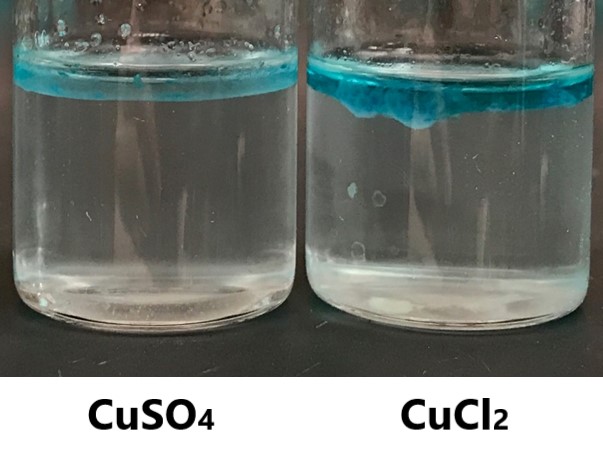


**Figure SI.11** Bottle tests conducted using 0.03 M CuCl_2_ (right) and CuSO_4_ (left), with 50:50 dioxane:water (v/v), at circum-neutral pH.


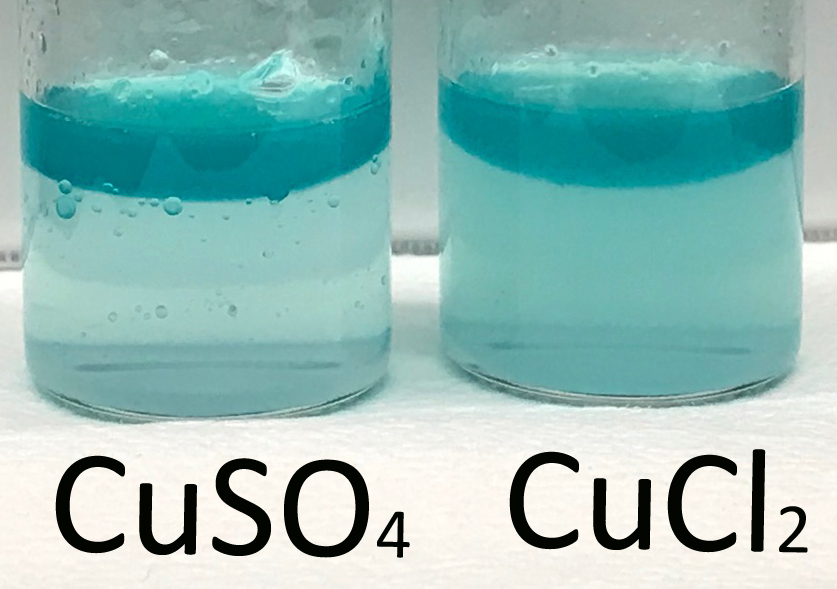


**Figure SI.12** Bottle tests conducted using 30 mM CuCl_2_ or CuSO_4_ solutions (70% v/v), toluene (10% v/v) and lauric acid in canola oil (10% v/v). Similar results were obtained without canola oil (Fig. SI.13).


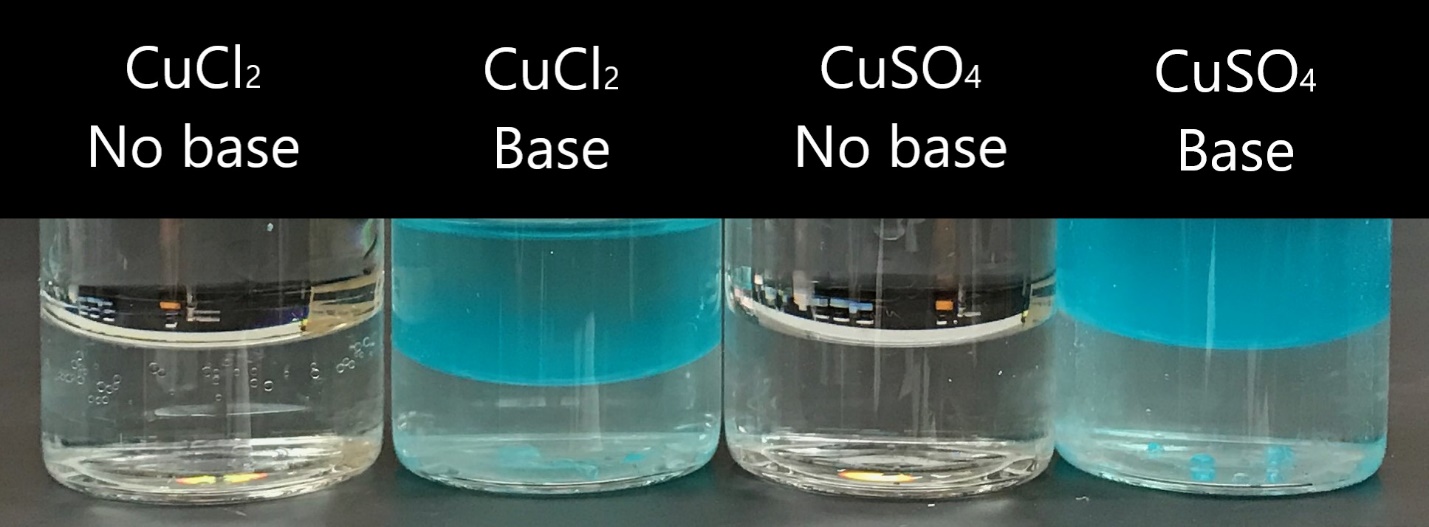


**Figure SI.13** Bottle tests conducted using 30 mM CuCl_2_ or CuSO_4_ solutions, using 0.125 M lauric acid and 50:50 toluene:water (v/v). Water was either at circum-neutral pH or at basic pH. Images were taken one day after hand-shaking the vials.


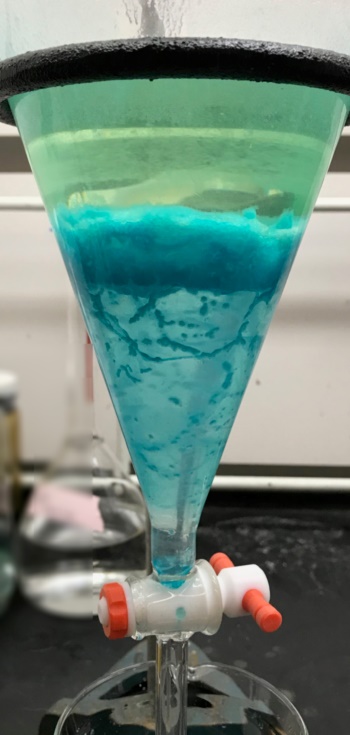


**Figure SI.14** Separation between the oil phase (10% v/v toluene and 10% v/v 0.25 M lauric acid in canola oil) and the water layer. The aqueous phase (80 %, v/v) initially contained 30 mM CuCl_2_. Similar results were obtained with 30 mM CuSO_4_. Note the segregation of flocs at the toluene-water interface.


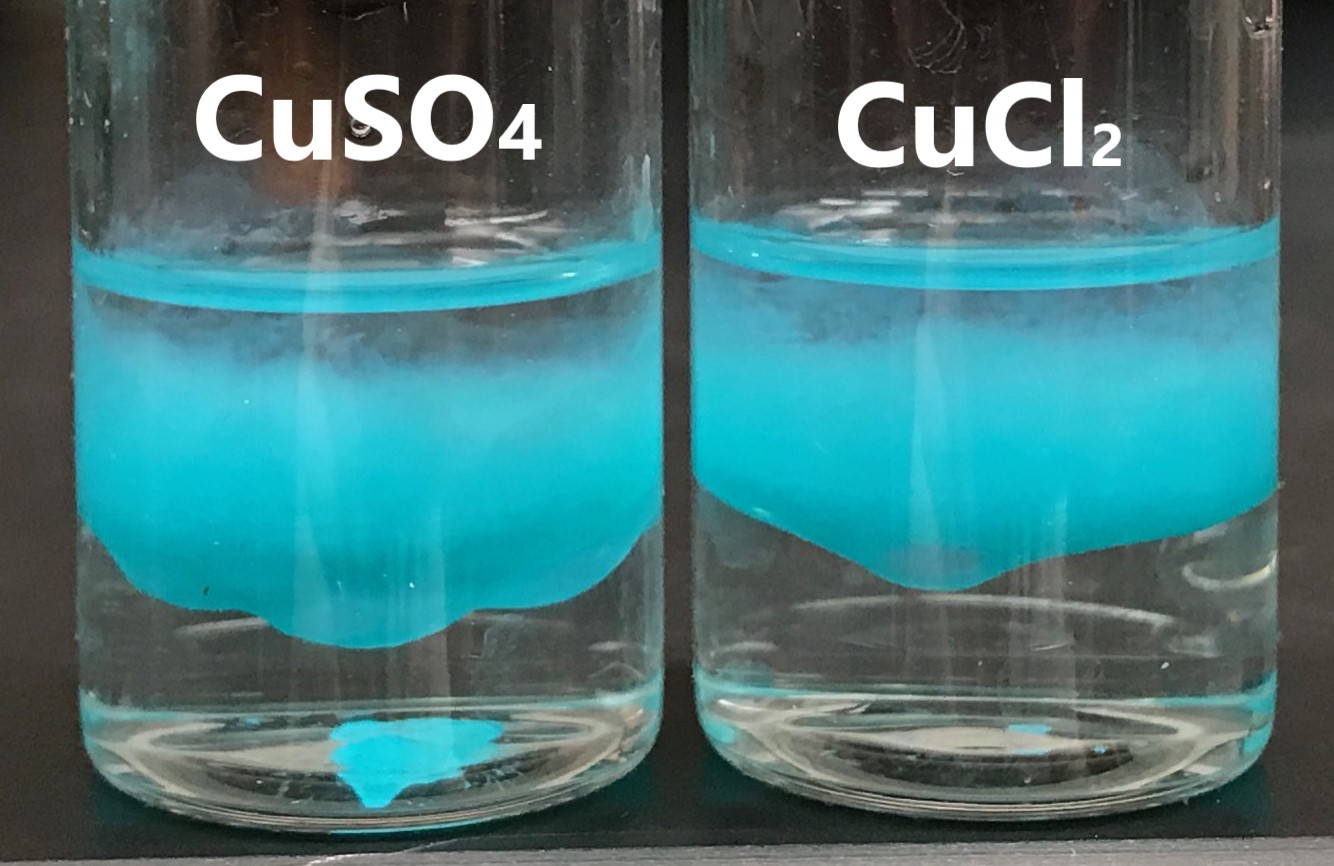


**Figure SI.15** Bottle tests conducted using 30 mM CuCl_2_ or CuSO_4_ aqueous solutions (50% v/v relative to hexane), hexane, and 0.125 M lauric acid.


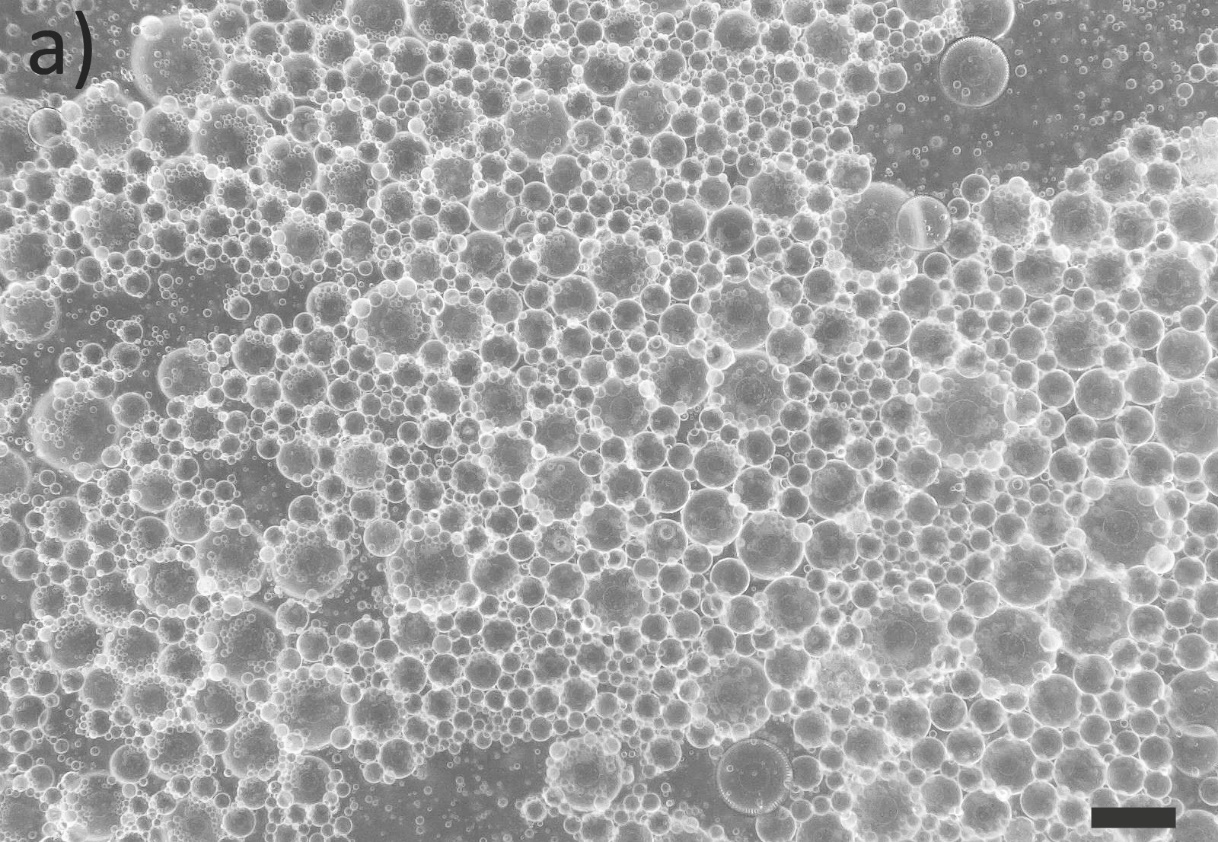

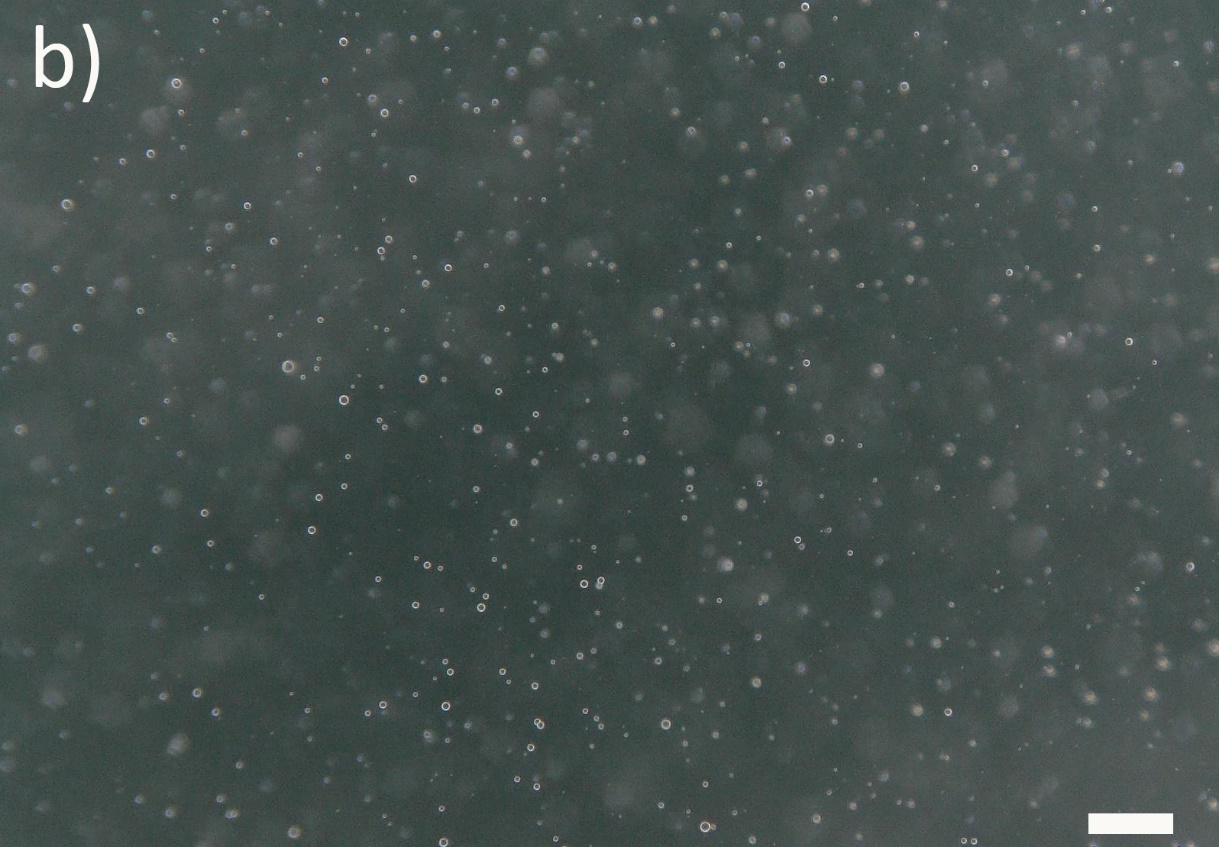


**Figure SI.16** Optical microscopy images of water in oil (a) and oil in water (b) emulsions of samples prepared using toluene (10% v/v), 0.25 M lauric acid in canola oil (10% v/v), and 10 mM NaOH (relative to the water phase). The scalebar is 100 μm.


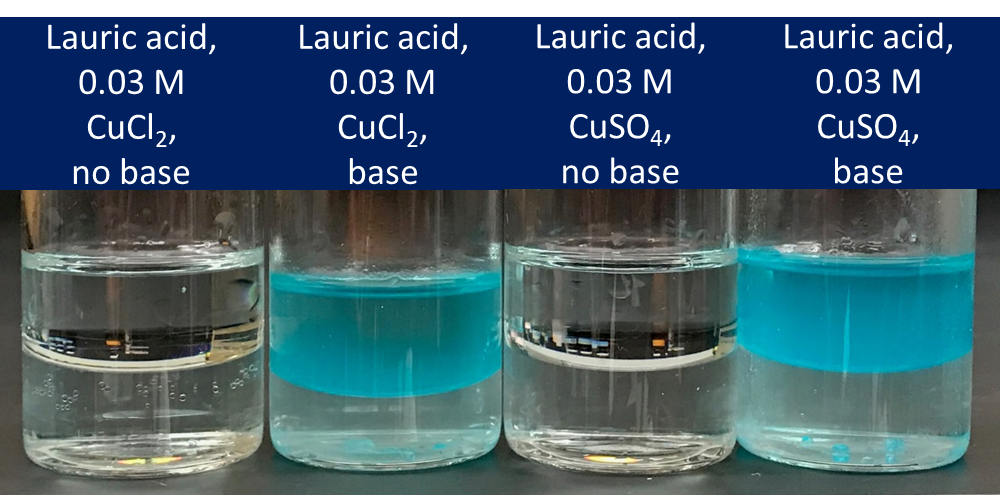


**Figure SI.17** Emulsions obtained using toluene and aqueous solutions of either 0.03 M CuSO_4_ or CuCl_2_. The toluene:water ratio is 50:50, and the lauric acid concentration is 0.125 M. Vials were equilibrated overnight after hand-shaking.

1. **Gaussian Fit Paramters**

**Table SI.4** Parameters for the Gaussian fits to XRD patterns in the SAXS region.

| Sample (THF % relative to water, v/v) | 100% | 95%, neutral | 90%, neutral | 90%, acidic |
| --- | --- | --- | --- | --- |
| mean | 0.05729 | 0.05621 | 0.05610 | 0.05469 |
| standard deviation | 0.03887 | 0.03644 | 0.03439 | 0.03521 |
| 95% confidence interval (mean) | 0.05718 to 0.05740 | 0.05613 to 0.05630 | 0.05602 to 0.05618 | 0.05461 to 0.05478 |
| 95% confidence interval (standard deviation) | 0.03877 to 0.03897 | 0.03636 to 0.03651 | 0.03431 to 0.03446 | 0.03514 to 0.03529 |
| R^2^ | 0.9993 | 0.9996 | 0.9996 | 0.9996 |
| Number of points analyzed | 1247 | 1167 | 1047 | 1047 |

**References**

[1] L. Ning, W. De-Ning, Y. Sheng-Kang, Hydrogen bonding between urethane and urea: band assignment for the carbonyl region of FTi. r. spectrum, Polymer, 37 (1996) 3045-3047.

[2] P.W. Stott, A.C. Williams, B.W. Barry, Mechanistic study into the enhanced transdermal permeation of a model β-blocker, propranolol, by fatty acids: a melting point depression effect, International journal of pharmaceutics, 219 (2001) 161-176.
